# IL-33/ST2 axis of human amnion fibroblasts participates in inflammatory reactions at parturition

**DOI:** 10.1186/s10020-023-00668-9

**Published:** 2023-07-04

**Authors:** Wen-jia Lei, Fan Zhang, Yi-kai Lin, Meng-die Li, Fan Pan, Kang Sun, Wang-sheng Wang

**Affiliations:** 1 Center for Reproductive Medicine, Ren Ji Hospital, School of Medicine, Shanghai Jiao Tong University, Shanghai, P. R. China; 2 Shanghai Key Laboratory for Assisted Reproduction and Reproductive Genetics, Shanghai, P. R. China

**Keywords:** IL-33, ST2, COX-2, Amnion, Parturition

## Abstract

**Background:**

Inflammation of the fetal membranes is an indispensable event of labor onset at both term and preterm birth. Interleukin-33 (IL-33) is known to participate in inflammation via ST2 (suppression of tumorigenicity 2) receptor as an inflammatory cytokine. However, it remains unknown whether IL-33/ST2 axis exists in human fetal membranes to promote inflammatory reactions in parturition.

**Methods:**

The presence of IL-33 and ST2 and their changes at parturition were examined with transcriptomic sequencing, quantitative real-time polymerase chain reaction, Western blotting or immunohistochemistry in human amnion obtained from term and preterm birth with or without labor. Cultured primary human amnion fibroblasts were utilized to investigate the regulation and the role of IL-33/ST2 axis in the inflammation reactions. A mouse model was used to further study the role of IL-33 in parturition.

**Results:**

Although IL-33 and ST2 expression were detected in both epithelial and fibroblast cells of human amnion, they are more abundant in amnion fibroblasts. Their abundance increased significantly in the amnion at both term and preterm birth with labor. Lipopolysaccharide, serum amyloid A1 and IL-1β, the inflammatory mediators pertinent to labor onset, could all induce IL-33 expression through NF-κB activation in human amnion fibroblasts. In turn, via ST2 receptor, IL-33 induced the production of IL-1β, IL-6 and PGE2 in human amnion fibroblasts via the MAPKs-NF-κB pathway. Moreover, IL-33 administration induced preterm birth in mice.

**Conclusion:**

IL-33/ST2 axis is present in human amnion fibroblasts, which is activated in both term and preterm labor. Activation of this axis leads to increased production of inflammatory factors pertinent to parturition, and results in preterm birth. Targeting the IL-33/ST2 axis may have potential value in the treatment of preterm birth.

**Supplementary Information:**

The online version contains supplementary material available at 10.1186/s10020-023-00668-9.

## Background

Parturition is known to be an inflammatory process of intrauterine tissues, including the fetal membranes, decidua, and cervix, at both term and preterm irrespective of the presence or absence of infection (Challis et al. 2009; Christiaens et al. 2008; Lim et al. 2019; Menon et al. 2019; Romero et al. 2006). Increased production of pro-inflammatory cytokines in inflammatory intrauterine tissues can further intensify the inflammatory process and invoke the production of prostaglandins (PGs), particularly PGE2 and PGF2α, which in turn induce parturition by stimulating myometrial contraction, cervical ripening and membrane rupture, etc. (Farina et al. 2005; Gibb 1998; Li W. J. et al. 2021; Romero et al. 2014). Overwhelming evidence indicates that premature and exaggerated inflammation of intrauterine tissues can induce preterm birth, which is a leading cause of perinatal mortality and morbidity (Goldenberg et al. 2008; Liu et al. 2016). Of the intrauterine tissues, inflammation of the fetal membranes draws particular attention as chorioamnionitis, also known as intra-amniotic infection, which is a common cause of not only premature ruptures of the amniotic sac membranes but also preterm birth (Romero et al. 2014). Inflammation of the fetal membranes triggers the release of various inflammatory signaling molecules including prostaglandins, pro-inflammatory cytokines and metalloproteinases which are involved in parturition and membrane rupture (Menon et al. 2019, 2020). The amnion layer of the fetal membranes not only determines the tensile strength of the fetal membranes but also harbors a myriad of inflammatory signaling molecules (Menon et al. 2020; Oyen et al. 2006; Wang et al. 2018). We and others have demonstrated that mesenchymal cells of the amnion produce a large amount of prostaglandins and pro-inflammatory cytokines (Keelan et al. 1997, 2003; Li et al. 2017; Ni et al. 2022; Okazaki et al. 1981; Zhang et al. 2017). Therefore, understanding the expression and regulation of pro-inflammatory signaling molecules in amnion mesenchymal cells may help further understand the unresolved mechanisms of human parturition.

Interleukin-33 (IL-33) is a member of the IL-1 superfamily, which can function both as an alarmin cytokine and as a chromatin-associated nuclear factor regulating gene transcription (Bertheloot et al. 2017; Cayrol et al. 2018; 2022). As an alarmin cytokine, upon tissue injury or cell damage, IL-33 released into the extracellular environment interacts with its specific receptor ST2 (suppression of tumorigenicity 2, also known as IL1RL1), which exists in two differentially-spliced forms: the membrane-bound genuine ST2 receptor that mediates IL-33 cytokine activities and a soluble receptor (sST2) that inhibits IL-33 bioactivities (Griesenauer et al. 2017). The membrane-bound ST2 is a member of the Toll-like receptor/IL1R superfamily (Chackerian et al. 2007; Schmitz et al. 2005), which can activate intracellular signaling pathways including nuclear factor-kappa B (NF-κB) and mitogen-activated protein kinase (MAPK) pathways to participate in both acute and chronic inflammatory diseases (Huang et al. 2021; Huang S. J. et al. 2018; Miller A. M. 2011; Milovanovic et al. 2012; Vasanthakumar et al. 2019). It is known that IL-33 and ST2 are expressed in a variety of cell types, including immunocytes as well as endothelial cells, epithelial cells and mesenchymal cells (Chen H. et al. 2018; Hu et al. 2014; Miller J. E. et al. 2022; Moussion et al. 2008). However, it is unknown whether amnion mesenchymal cells, the reservoir of multiple inflammatory signaling molecules, can also express IL-33 and ST2 to participate in parturition.

In this study, we addressed this issue by examining cell-specific expression patterns of IL-33 and ST2 in human amnion and their changes in the amnion at term and preterm birth. Furthermore, we investigated the effect of bacterial product lipopolysaccharide (LPS), acute-phase protein serum amyloid A1 (SAA1), IL-1β, and IL-33 itself on IL-33 expression in human amnion fibroblasts. Moreover, the role of IL-33 and ST2 in the regulation of pivotal inflammatory molecules pertinent to parturition, such as IL-1β, IL-6, and cyclooxygenase-2 (COX-2), the rate-limiting enzyme involved in prostaglandin synthesis, and the underlying intracellular signaling pathways were also investigated in human amnion fibroblasts. Finally, the parturition-initiating effect of IL-33 was examined in pregnant mice.

## Methods

### Collection of human fetal membranes

Human fetal membranes were collected from uncomplicated pregnancies with written informed consent under a protocol approved by the Ethics Committee of Ren Ji Hospital, Shanghai Jiao Tong University School of Medicine. Participating subjects were divided into four groups: elective cesarean section without labor at term (designated as term non-labor, TNL); spontaneous labor at term (designated as term labor, TL); preterm elective cesarean section without labor for maternal or fetal conditions including placenta previa, vasa previa, and fetal distress (designated as preterm non-labor, PNL); and spontaneous preterm labor with no indication of infection (designated as preterm labor, PL). Pregnancies with complications such as preeclampsia, fetal growth restriction, gestational diabetes, and chorioamnionitis were excluded from the study. Upon deliveries, the amnion layer at spontaneous (TL and PL) or artificial (TNL and PNL) rupture sites were peeled off the fetal membranes for subsequent studies. The entire amnion layer from the reflected membranes obtained from TNL was used for the preparation of amnion epithelial and fibroblast cells. The demographic features of recruited women are listed in Table 1.

**Table 1:** Demographic and clinical characteristics of recruited pregnant women

Demographic features	TNL (n = 8)	TL (n = 9)	*p-value* (TL vs. TNL)	PNL (n = 8)	PL (n = 11)	*p-value* (PL vs. PNL)
Maternal age (years)	32.43 ± 1.172	31.29 ± 0.8081	0.4378	34.38 ± 1.880	30.18 ± 1.102	0.0568
Gravidity	2(1–5)	1 (1–2)	0.0737	2.5(1–5)	1(1–5)	0.1418
Parity	1 (0–2)	1 (0–2)	0.2046	1(1–2)	1(1–1)	0.0578
Gestational age (weeks)	39.1 ± 0.1	39.2 ± 0.3	0.6776	34.6 ± 0.3	34.0 ± 0.48	0.2488
Gender (Male/female)	3/5	4/5	0.7715	4/4	4/7	0.6577
Birth weight (g)	3400 ± 90.73	3226 ± 110.5	0.2455	2381 ± 81.18	2313 ± 113.2	0.6573

Maternal age, gestational age, and birth weight were expressed as mean ± SEM and analyzed with unpaired Student’s t-test. Gravidity and parity were expressed as median (min-max) and analyzed with the Mann–Whitney U test. Gender was analyzed with Chi-Square test. Significance was set at P < 0.05

### Transcriptomic sequencing

Total RNA was extracted from amnion tissue obtained from TNL and TL (n = 3 each) using an RNA extraction kit (OMEGA Bio-Tek, Norcross, GA) following a protocol provided by the manufacturer. After extraction, RNA purity and integrity were determined using a NanoDrop ND-2000 and an Agilent 2200 TapeStation with the following parameters: A260/A280 ratio ≥ 1.8, A260/ A230 ratio ≥ 2.0 and RNA integrity number value ≥ 7.0. RNA-sequence libraries were constructed using a TruSeq RNA sample preparation kit (Illumina, San Diego, CA) following the manufacturer’s protocol. Sequencing of the libraries was performed on an IlluminaHiSeqTM 2500 system. A computational pipeline was used to process the RNA-sequence data. Reads were aligned to human genome [National Center for Biotechnology Information (NCBI); GRCh38] using Hisat v.2.1.0 with default options. The fragments per kilo base per million mapped reads (FPKM) were obtained. RNA sequencing data have been uploaded to NCBI with the Gene Expression Omnibus (GEO) accession number GSE166453 (Lu et al. 2021).

### Measurement of IL-33 and ST2 abundance in human amnion with quantitative real-time polymerase chain reaction (qRT-PCR) and western blotting

To further analyze the changes of IL-33 and ST2 abundance in human amnion in term and preterm labor, the amnion layer at the artificial rupture site (TNL and PNL) and spontaneous rupture site (TL and PL) was collected and snap-frozen in liquid nitrogen for total RNA and protein extraction.

Total RNA was extracted from the snap-frozen amnion tissue with a total RNA isolation Kit (Foregene, Chengdu, China). After examination of RNA quality and concentration, reverse transcription was carried out using a Prime-Script RT Master Mix Kit (TaKaRa, Kyoto, Japan). The amount of *IL33* and *IL1RL1* (ST2) mRNA was determined with qRT-PCR using the above reverse-transcribed cDNA and power SYBR® Premix Ex Taq™ (TaKaRa). Housekeeping gene *ACTB* (β-actin) was amplified in parallel for internal loading control. The relative mRNA abundance was quantitated using the 2-^ΔΔCt^ method. The primer sequences used for qRT-PCR are illustrated in Table S1.

Total cellular protein was extracted from the snap-frozen amnion tissue with an ice-cold radioimmunoprecipitation assay (RIPA) lysis buffer (Active Motif, Carlsbad, CA) containing inhibitors for protease and phosphatase (Roche, Indianapolis, IN). After determination of protein concentration using the Bradford method, the abundance of IL-33 and ST2 protein in each sample was determined with Western blotting. Briefly, 30 µg of protein from each sample was electrophoresed in a sodium dodecyl sulfate-polyacrylamide gel. After transferring to a nitrocellulose membrane blot, the blot was blocked with 5% non-fat milk and incubated with antibodies against IL-33 (1:1000; GeneTex, San Antonio, TX; #31,317) and ST2 (1;1000; PTMab, Hangzhou, China; #PTM-6483) overnight at 4 °C, followed by incubation with corresponding secondary antibodies conjugated with horseradish peroxidase. The peroxidase activity was developed with a chemiluminescence detection system (Millipore, Billerica, MA), which was visualized using a G-Box chemiluminescence image capture system (Syngene, Cambridge, U.K.). Internal loading control was performed by probing the same blot with antibodies against β-actin (1:10000; Proteintech, Wuhan, China; #60008-1) or glyceraldehyde 3-phosphate dehydrogenase (GAPDH; 1:10000; Proteintech; #60004-1). Since both β-actin and GAPDH were constantly expressed across samples (Supplementary Figure S1), the ratio of the band intensity of IL-33 or ST2 to that of β-actin was used to indicate protein abundance.

### Immunohistochemical staining of IL-33 and ST2 in human amnion tissue

To observe the distribution of IL-33 and ST2 in human amnion tissue, the amnion tissue from TL and PL was fixed in 10% paraformaldehyde and embedded in paraffin for sectioning for immunohistochemical staining. After deparaffinization and quenching the endogenous peroxidase activity with 0.3% H_2_O_2,_ the section was blocked with the non-immune serum, followed by incubation with the primary antibody against IL-33 (1:100; GeneTex; #31,317) or ST2 (1:100; R&D systems, Minneapolis, MN; #AF523-SP) or corresponding non-immune serum (Proteintech) for negative control overnight at 4 °C. After washing, a biotinylated secondary antibody and the avidin-biotin complex conjugated with horseradish peroxidase (Vector Laboratories, Burlingame, CA) were applied to the section for further incubation. The peroxidase activity was developed as a red color using the substrate 3-amino-9-ethyl carbazole (Vector Laboratories). The slide was counterstained with hematoxylin (blue color) and examined under a regular bright field microscope (Zeiss, Oberkochen, Germany).

### Preparation of primary human amnion fibroblasts and epithelial cells

Amnion fibroblasts and epithelial cells were isolated from the TNL amnion as described previously (Zhang et al. 2022a). Briefly, the amnion tissue was digested twice with 0.125% trypsin (Life Technologies Inc., Grand Island, NY) and then washed thoroughly with phosphate buffer saline (PBS). The epithelial cells in trypsin-digested medium and PBS wash were collected by centrifugation. The remaining amnion tissue was further digested with 0.1% collagenase (Sigma, St. Louis, MO) to release fibroblasts. Fibroblasts in the digestion medium were collected by centrifugation. Isolated fibroblasts and epithelial cells were cultured in Dulbecco’s Modified Eagle Medium (DMEM) containing 10% fetal bovine serum (FBS) and antibiotics (all from Life Technologies Inc.) at 37℃ in 5% CO_2_/95% air. This method of amnion cell isolation yields high purity of epithelial cells (> 99%) and fibroblasts (> 95%), which have been previously characterized with staining of cytokeratin-7 and vimentin, markers for epithelial and mesenchymal cells respectively (Mi et al. 2018).

### Treatment of human amnion fibroblasts

To compare the abundance of IL-33 and ST2 between amnion fibroblasts and epithelial cells, the cells were cultured for 3 days and then subjected to RNA and protein extraction for analysis with qRT-PCR and Western blotting respectively.

To study the regulation of IL-33 expression and the effect of IL-33 on inflammatory molecules, amnion fibroblasts were cultured for 3 days before reagent treatment in phenol red- and FBS-free DMEM. To examine the regulation of IL-33 expression in amnion fibroblasts, the cells were treated with IL-1β (0.1, 1, 10 ng/mL; Sigma; 24 h), LPS (1, 10, 50 ng/mL; Sigma; 24 h), SAA1 (1, 10, 50 ng/mL; PeproTech Inc., Rocky Hill, NJ; 24 h) and IL-33 (50, 100, 200 ng/mL; 8 h). To investigate the role of NF-κB in the regulation of IL-33 expression, the cells were treated with IL-1β (10 ng/mL; 24 h), LPS (10 ng/mL; 24 h), SAA1 (50 ng/mL; 24 h) and IL-33 (100 ng/mL; 8 h) in the presence or absence of JSH-23 (10 µM; Selleck, Houston, TX), an inhibitor of NF-κB nuclear translocation. To investigate the effect of IL-33 on the expression of COX-2, IL-1β and IL-6, time course and concentration-dependent studies were conducted. For time course study, fibroblasts were treated with IL-33 (100 ng/mL) for 2, 4, 8, 12, 24 h. For concentration-dependent study, fibroblasts were treated with IL-33 for 8 h at concentrations of 50, 100 and 200 ng/mL. To examine the effects of IL-33 (100 ng/mL) on the phosphorylation of MAPKs (p38, ERK1/2 and JNK) and p65, a subunit of NF-κB, a time course study (0.5, 1, 2, 4 and 6 h) was carried out. To investigate the involvement of MAPKs pathway in the phosphorylation of p65 and the regulation of COX-2, IL-1β and IL-6 by IL-33, fibroblasts were treated with IL-33 (100 ng/mL) for 8 h in the presence or absence of the p38 inhibitor SB203580 (10 µM; Selleck), the ERK1/2 inhibitor PD98059 (20 µM; Selleck) or the JNK inhibitor SP600125 (10 µM; Selleck). To study the involvement of NF-κB in the induction of COX-2, IL-1β and IL-6 by IL-33, amnion fibroblasts were treated with IL-33 (100 ng/mL) for 8 h in the presence or absence of JSH-23 (10 µM). To explore the involvement of ST2 in the inactivation of MAPKs and NF-κB, and in the induction of COX-2, IL-1β and IL-6 expression by IL-33, amnion fibroblasts were treated with IL-33 (100 ng/mL) in the presence or absence of small interfering RNA (siRNA)-mediated knockdown of ST2. The method of siRNA transfection is described below. All inhibitors were added 1 h before IL-1β, LPS, SAA1 or IL-33 treatment. After treatments, the conditioned culture medium was collected for the measurement of secreted IL-1β, IL-6 and PGE2 with enzyme immunoassay (ELISA) kits (PGE2, Cayman Chemical, Ann Arbor, MI; IL-1β and IL-6, Proteintech) according to the protocols provided by the manufacturers. IL-33 in the conditioned medium was measured with Western blotting after concentrating with a centrifugal filter device (Millipore) following a manufacturers’ protocol as described previously (Wang W. S. et al. 2019). The cells were processed for the extraction of total RNA and cellular protein for analyses of COX-2, IL-1β, IL-6 or IL-33 mRNA or protein abundance with qRT-PCR and Western blotting respectively.

### Transfection of siRNA in human amnion fibroblasts with electroporation

To study the role of the ST2 in the mediation of IL-33 effects, siRNA-mediated knockdown of ST2 was performed. Briefly, after isolation, amnion fibroblasts were transfected with 50 nM of two sets of siRNA against ST2 (1#: 5’-CTCTGTTTCCAGTAATCGGAGCC-3’; 2#: 5’- GCAGCCAAGAACTGAGTGCCTT-3’; GenePharma, Shanghai, China) or randomly scrambled siRNA (5′-UUCUCCGAACGUGUCACGUTT-3′) in Opti-MEM (Life Technologies Inc.) using an electroporator (Nepa Gene, Chiba, Japan) at 165 V for 5ms. The cells were then incubated in DMEM containing 10% FBS for 72 h before IL-33 treatment. The knockdown efficiency was assessed with qRT-PCR and Western blotting.

### RNA and protein extraction from human amnion fibroblasts for analysis with qRT-PCR and western blotting

Total RNA was extracted from the above-treated amnion fibroblasts using a total RNA isolation kit (Foregene, Chengdu, China). The mRNA abundance of *PTGS2* (COX-2), *IL1B*, *IL6* and *IL33* was measured with qRT-PCR as described above. The housekeeping gene *GAPDH* was amplified in parallel as an internal loading control. The primers sequences used in qRT-PCR are illustrated in the Table S1. Cellular protein was extracted with the RIPA lysis buffer (Active Motif) containing inhibitors for proteases and phosphatase (Roche). The protein abundance of COX-2, IL-33, ST2, total p38, phosphorylated p38 (p-p38) at Thr180/Tyr182, total ERK, phosphorylated ERK (p-ERK) at Thr202/Tyr204, total JNK, phosphorylated JNK (p-JNK) at Thr183/Tyr185, total p65, phosphorylated p65 (p-p65) at Ser536 was measured with Western blotting as described above. Internal loading controls were performed by either probing the blot with a GAPDH antibody for the cellular protein or Ponceau S staining for the concentrated conditioned culture medium. Ratio of the phosphorylated protein over its corresponding total protein was used to indicate the relative abundance of phospho-proteins. The primary antibodies used for Western blotting in this study were as follows: ST2 (1;1000; PTMab; #PTM-6483), p38 (1:500; #8690), phosphorylated p38 at Thr180/Tyr182 (1:500; #4511), ERK1/2 (1:500; #4631), phosphorylated ERK1/2 at Thr202/Tyr204 (1:500; #4370), JNK (1:1000; #9252), phosphorylated JNK at Thr183/Tyr185 (1:1000; #9255), NF-κB p65 (1:1000; #6956), phosphorylated NF-κB p65 (1:1000; #3033S), PTGS2 (1:1000; #12,282) (all from Cell Signaling, Danvers, MA), IL-33 (1:1000; GeneTex; #31,317) and GAPDH antibody (1:10000; Proteintech; #60004-1).

### Animal study

Animal experimentation was conducted following ARRIVE guidelines for animal care which was approved by the Institutional Review Board of Ren Ji Hospital, Shanghai Jiao Tong University School of Medicine. C57BL/6 mice (Ziyuan, Hangzhou, China) aged from 10 to 13 weeks were mated overnight. The presence of a vaginal plug in the morning was counted as gestational day 0.5. A total of 29 pregnant mice were used in this study. To observe whether IL-33 can induce preterm birth, IL-33 (1 µg per dam) was injected intraperitoneally on gestational days 16.5 and 17.5. An equal volume of PBS served as control. 17 pregnant mice were allowed to deliver spontaneously for observation of delivery time, and the other 12 pregnant mice were sacrificed 6 h following injection of IL-33 or PBS on gestational days 17.5 for collection of fetal membranes to examine the abundance of COX-2, IL-1β and IL-6 after frozen in liquid nitrogen for protein extraction with Western blotting or ELISA.

### Statistical analysis

All data are reported as means ± SEM. The number for each experiment indicates repeated experiments using different amnion tissue collected from independent patients or animals. After normality examination with the Shapiro-Wilk test, paired Student’s t-test or Mann-Whitney U test or one-way ANOVA test followed by the Newman-Keuls Multiple Comparison Test was performed where appropriate. Statistical significance was defined as P < 0.05.

## Results

### Expression of IL-33 and ST2 in human amnion at parturition

**Fig. 1:**
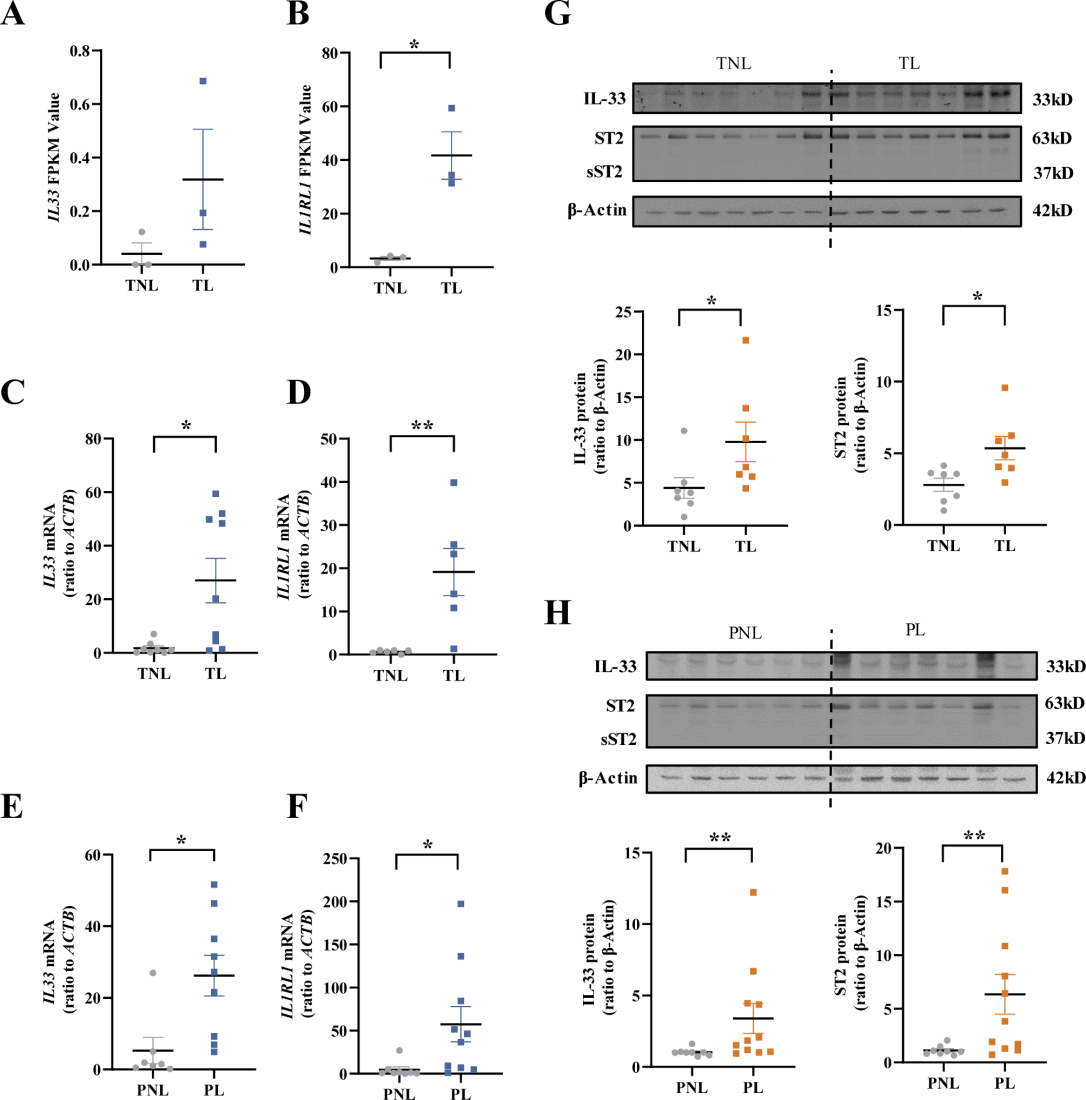
Expression of IL-33 and ST2 in human amnion at term and preterm labor. (**A** and **B**) Abundance of *IL33* and *IL1RL1* gene transcripts in the amnion in the TL (term labor, n = 3) and TNL (term non-labor, n = 3) groups as revealed by transcriptomic sequencing. FPKM, fragments per kilo base per million mapped reads. **(C and D)** Abundance of *IL33* and *IL1RL1* mRNA in the amnion of the TL (n = 9) and TNL (n = 8) groups as measured with qRT-PCR. **(E and F)** Abundance of *IL33* and *IL1RL1* mRNA in the amnion of the PL (preterm labor, n = 9) and PNL (preterm non-labor, n = 7) groups as measured with qRT-PCR. (**G**) Abundance of IL-33 and ST2 protein in the amnion of the TL (n = 7) and TNL (n = 7) groups as measured with Western blotting. (**H**) Abundance of IL-33 and ST2 protein in the amnion of the PL (n = 11) and PNL (n = 8) groups as measured with Western blotting. Top panels are the representative immunoblots. Data are mean ± SEM. Statistical analysis was performed with the Mann–Whitney U test. *p < 0.05, **p < 0.01 vs. TNL or PNL

Analysis of the transcriptomic sequencing data (NCBI GEO, accession number GSE166453) (Lu et al. 2021) revealed the existence of both *IL33* and *ST2* (*IL1RL1*) transcripts in human amnion (Fig. 1A and B). Moreover, the abundance of *IL33* and *ST2* transcripts was significantly increased in human amnion in the TL group as compared to the TNL group (Fig. 1A and B). Assay with qRT-PCR not only confirmed the increases in *IL33* and *IL1RL1* mRNA abundance in human amnion in the TL group but also their increases in the PL group as compared to the PNL group (Fig. 1C-F). Consistently, the protein abundance of IL-33 and ST2 was also significantly increased in human amnion in both TL and PL groups when compared with those in TNL and PNL groups respectively (Fig. 1G and H). However, sST2 protein was not detected in human amnion (Fig. 1G and H). We also compared the abundance of IL-33 and ST2 between TL and PL groups, and found no significant changes between these two groups (Supplementary Figure S2). These findings suggest that IL-33 and ST2 are present in human amnion, and there may be activation of IL-33/ST2 axis in human amnion in both term and preterm labor.

### Distribution of IL33 and ST2 in human amnion

**Fig. 2:**
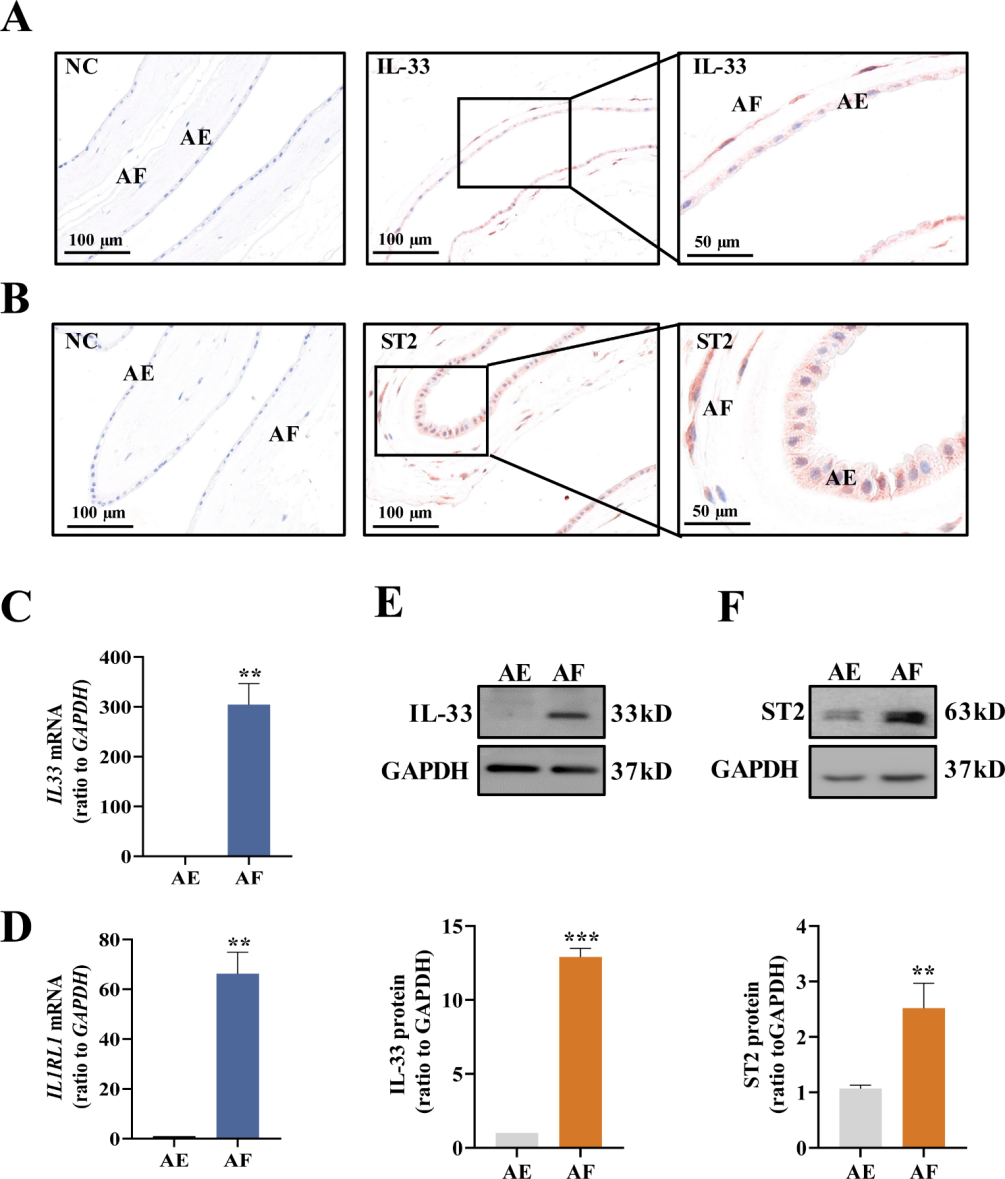
Distribution of IL-33 and ST2 in human amnion tissue. (**A** and **B**) Immunohistochemical staining of IL-33 (**A**) and ST2 (**B**) in human amnion showing the presence of IL-33 and ST2 in both epithelial cells and fibroblasts. (**C and D**) Comparison of *IL33* and *IL1RL1* mRNA abundance in human amnion fibroblasts and epithelial cells as determined with qRT-PCR. n = 3. (**E and F**) Comparison of IL-33 and ST2 protein abundance in human amnion fibroblasts and epithelial cells as determined with Western blotting. n = 3. The top panels of E and F are the representative immunoblots and the bottom panels of E and F are the average data. AE, amnion epithelial cells; AF, amnion fibroblasts. Data are mean ± SEM. Statistical analysis was performed with paired Student’s t-test. **p < 0.01, ***p < 0.001 vs. AE

Immunohistochemical staining of human amnion tissue collected from TL and PL showed that IL-33 and ST2 staining was detected in both mesenchymal fibroblasts and epithelial cells (Fig. 2A and B, Supplementary Figure S3). Measurements with qRT-PCR and Western blotting showed that the abundance of *IL33* and *ST2* mRNA and protein was significantly higher in amnion fibroblasts than epithelial cells (Fig. 2C-F), suggesting that amnion fibroblasts are the primary site of IL-33 and ST2 expression in human amnion.

### Induction of IL-33 expression by inflammatory mediators in human amnion fibroblasts

**Fig. 3:**
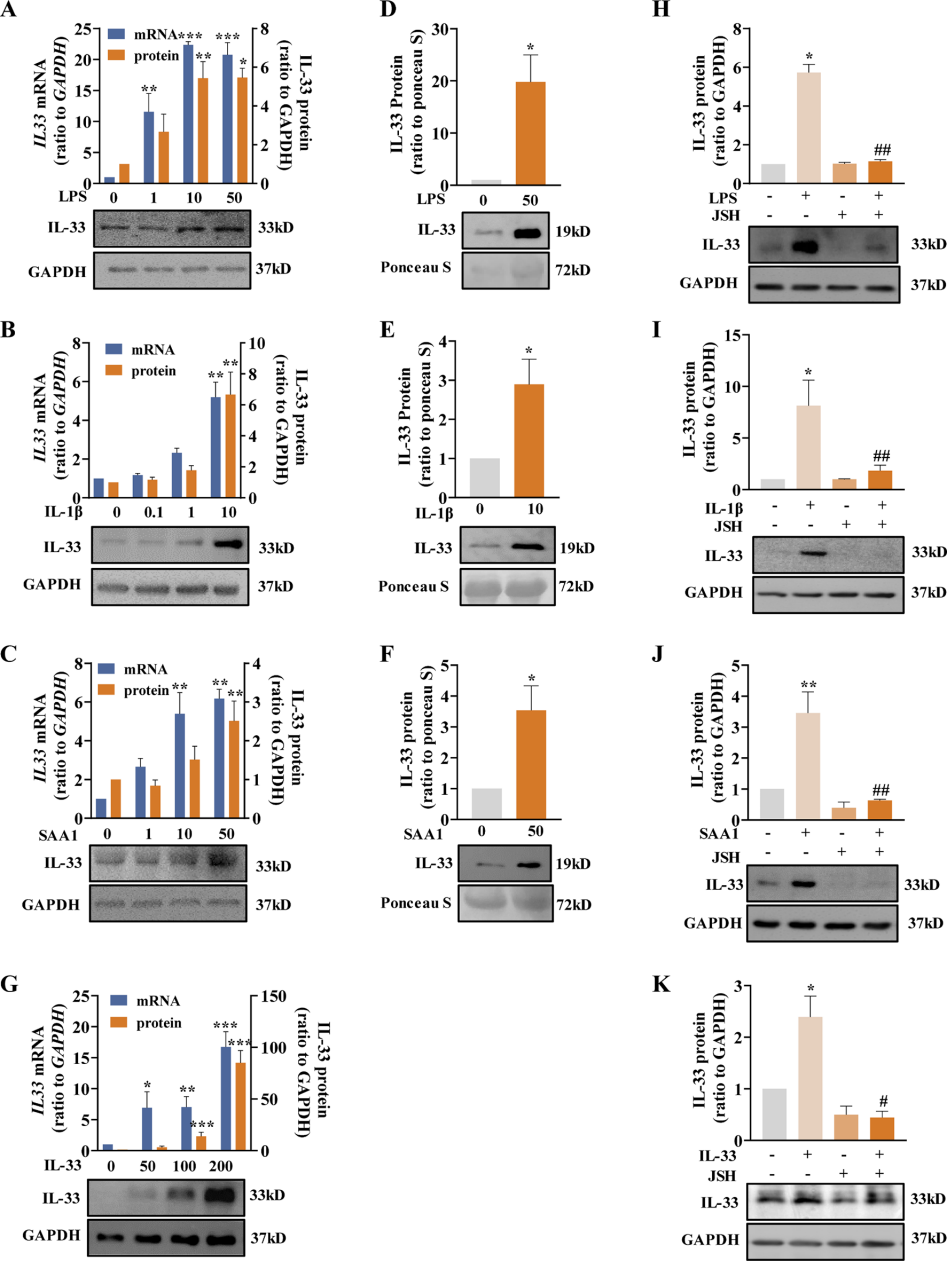
Induction of IL-33 by LPS, SAA1, IL-1β and IL-33 in human amnion fibroblasts. (**A**-**C**) Concentration-dependent effects of LPS (1, 10 and 50 ng/mL; 24 h; A), IL-1β (0.1, 1 and 10 ng/mL; 24 h; B) and SAA1 (1, 10 and 50 ng/mL; 24 h; C) on *IL33* mRNA and protein abundance in amnion fibroblasts. n = 3–4. (**D-F**) Effects of LPS (50 ng/mL; 24 h; D), IL-1β (10 ng/mL; 24 h; E) and SAA1 (50 ng/mL; 24 h; F) on IL-33 secretion. n = 3–4. (**G**) Concentration-dependent effect of IL-33 (50, 100 and 200 ng/mL; 8 h) on *IL33* mRNA and protein abundance in amnion fibroblasts. n = 3–4. (**H-K**) Blockade of LPS (10 ng/mL; 24 h)-, IL-1β (10 ng/mL; 24 h)-, SAA1 (50 ng/mL; 24 h)- and IL-33 (100 ng/mL; 8 h)-induced increases in IL-33 protein abundance by NF-κB inhibitor JSH-23 (JSH; 10 µM). n = 3. Bottom panels are the representative immunoblots and top panels are the average data. Data are mean ± SEM. Statistical analysis was performed with one-way ANOVA test followed by the Newman-Keuls multiple comparison test (**A-C**, **G-K**) or paired Student’s t-test (**D-F**). *p < 0.05, **p < 0.01, ***p < 0.001 vs. 0 ng/mL; #p < 0.05, ##p < 0.01 vs. LPS-, IL-1β-, SAA1- or IL-33-treated groups

We next examined the upstream signals that may regulate IL-33 expression in human amnion at parturition. We found that inflammatory mediators pertinent to parturition or preterm labor including LPS (1, 10, 50 ng/mL; 24 h), IL-1β (0.1, 1, 10 ng/mL; 24 h) and SAA1 (1, 10, 50 ng/mL; 24 h) (Keelan et al. 2003; Li et al. 2017; Ye et al. 2015), all increased *IL33* mRNA and protein abundance in a concentration-dependent manner in amnion fibroblasts (Fig. 3A-C). Moreover, IL-33 secretion was also significantly increased by LPS (50 ng/mL), SAA1 (50 ng/mL) and IL-1β (10 ng/mL) in amnion fibroblasts (Fig. 3D-F). Of interest, IL-33 (50, 100 and 200 ng/mL) also induced its own mRNA and protein in amnion fibroblasts in a concentration-dependent manner (Fig. 3G). Mechanistic studies showed that the induction of IL-33 expression by these inflammatory mediators including IL-33 itself could be completely blocked by JSH-23 (JSH; 10 µM), a blocker of NF-κB nuclear translocation (Fig. 3H-K). These data suggest that the common inflammatory transcription factor NF-κB is crucial for the regulation of IL-33 expression. The IL-33 self-induction property indicates that there may be a feed-forward induction of IL-33 expression in human amnion fibroblasts at parturition.

### Effect of IL-33 on inflammatory factors in human amnion fibroblasts

**Fig. 4:**
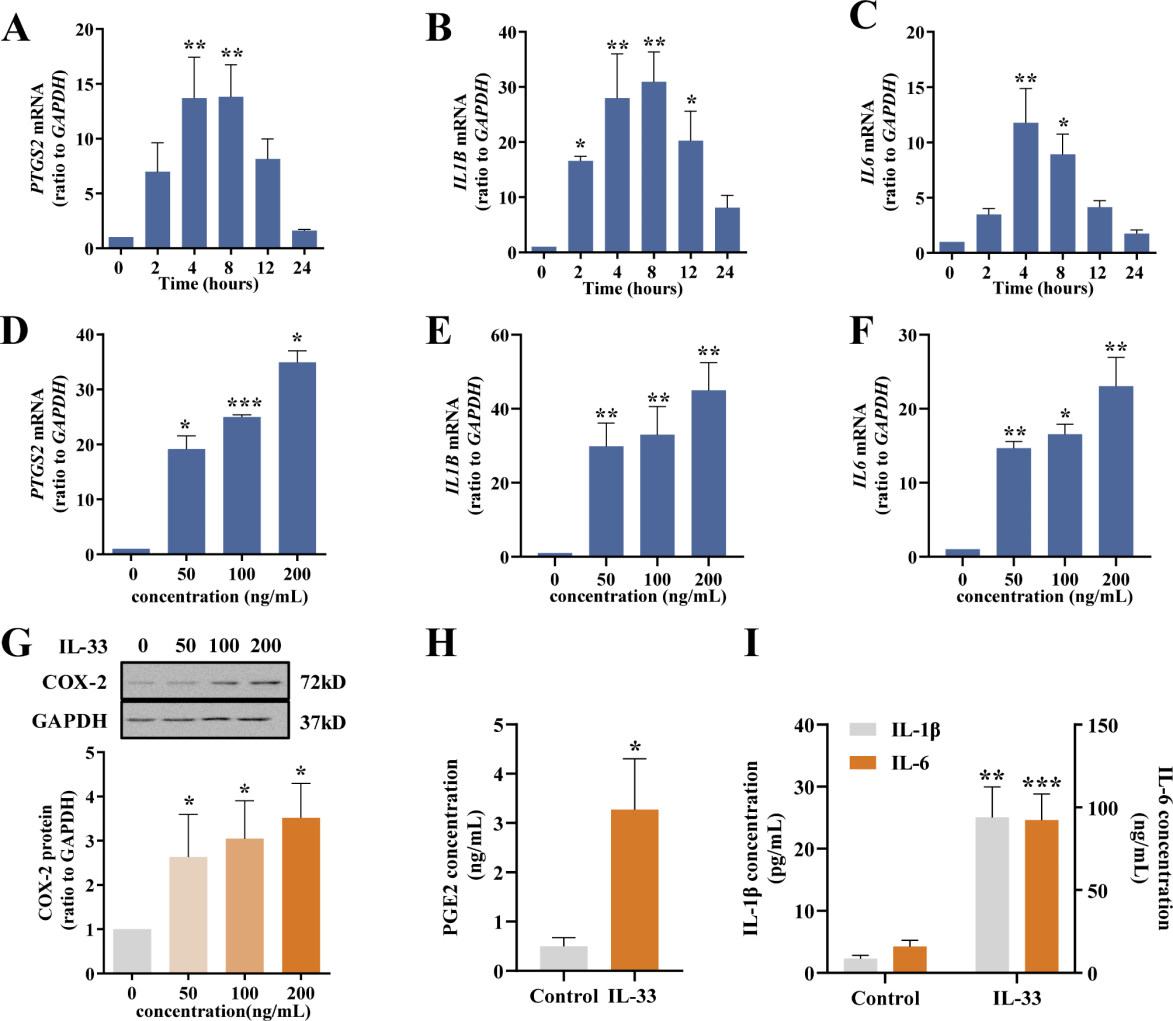
Effect of IL-33 on the expression of COX-2, IL-1β and IL-6 in human amnion fibroblasts. (**A-C**) Time-dependent (2, 4, 8, 12 and 24 h) induction of *PTGS2, IL1B* and *IL6* mRNA expression by IL-33 (100 ng/mL). n = 3. (**D-F**) Concentration-dependent induction of *PTGS2, IL1B* and *IL6* mRNA expression by IL-33 (50, 100 and 200 ng/mL; 8 h). n = 3. (**G**) Concentration-dependent induction of COX-2 protein by IL-33 (50, 100 and 200 ng/mL; 8 h). n = 4. The top panel is the representative immunoblots and the bottom panel is the average data. (**H and I**) Induction of PGE2 (**H**), IL-1β and IL-6 (**I**) secretion by IL-33 (100 ng/mL; 8 h). n = 7. Data are mean ± SEM. Statistical analysis was performed with one-way ANOVA test followed by the Newman-Keuls multiple comparison test (**A-G**) or paired Student’s t-test (**H** and **I**). *p < 0.05, **p < 0.01, ***p < 0.001 vs. 0 h or 0 ng/mL

Next, we explored whether IL-33 had any effect on the expression of inflammatory factors pertinent to parturition in human amnion fibroblasts, including IL-1β, IL-6 and COX-2, the rate-limiting enzyme in prostaglandin synthesis. Time course study showed that IL-33 (100 ng/mL) induced the genes encoding IL-1β, IL-6 and COX-2 in a time-dependent manner with maximal effects observed at 8 h for *PTGS2* and *IL1B*, and at 4 h for *IL6* (Fig. 4A-C). Concentration-dependent study revealed that IL-33 (50, 100 and 200 ng/mL) induced the genes encoding IL-1β, IL-6 and COX-2 in a dose-dependent manner with maximal effects observed at 200 ng/mL (Fig. 4D-F). In addition to the induction of gene expression, IL-33 also increased COX-2 protein abundance (Fig. 4G) and the secretion of PGE2, IL-1β and IL-6 in amnion fibroblasts (Fig. 4H and I).

### Roles of MAPKs and NF-κB in the induction of COX-2, IL-1β and IL-6 expression by IL-33 in human amnion fibroblasts

**Fig. 5:**
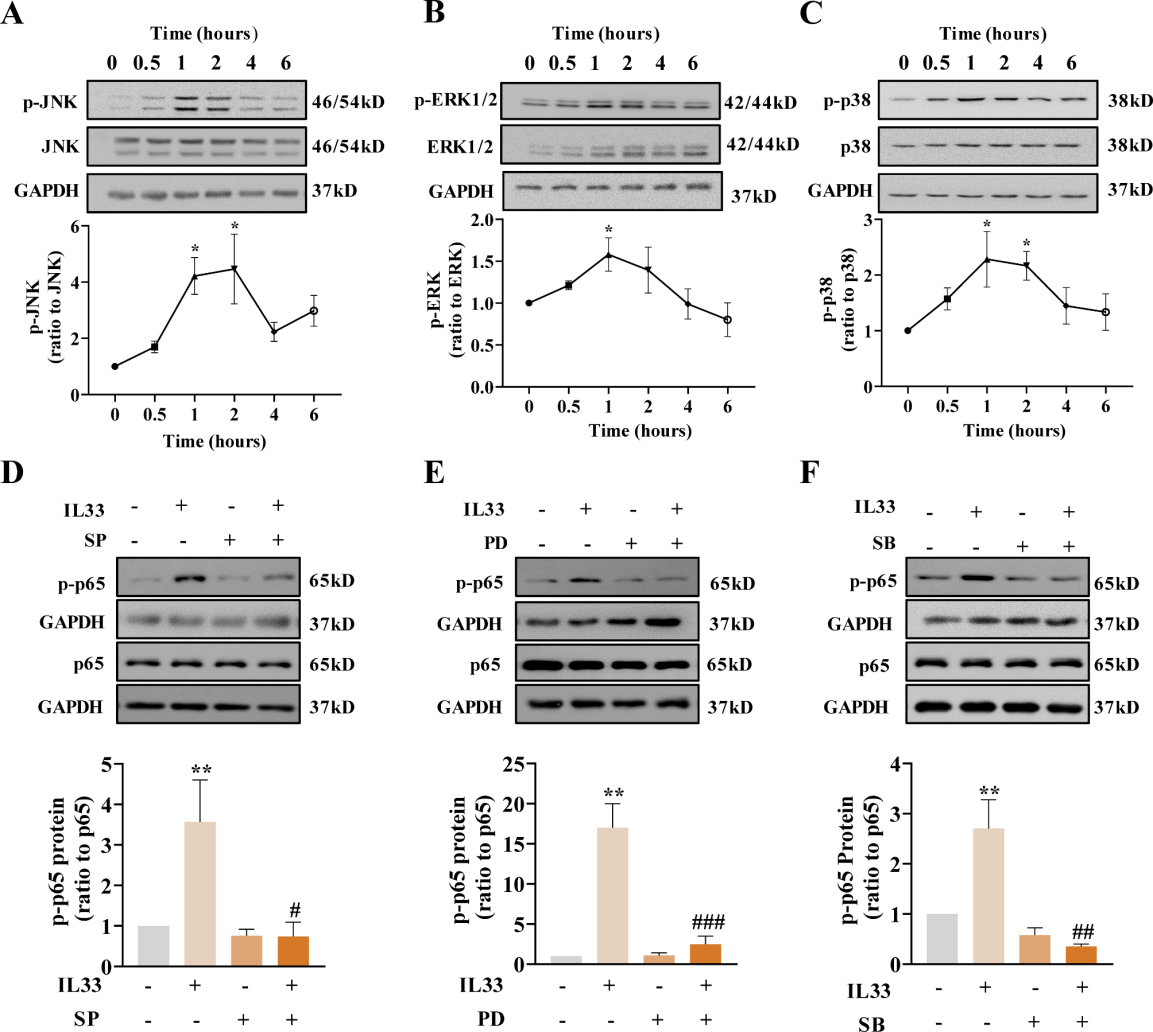
Phosphorylation of JNK, ERK1/2, p38 and NF-κB p65 by IL-33 in human amnion fibroblasts. (**A**–**C**) Time-dependent effects of IL-33 (100 ng/mL) on JNK (**A**), ERK1/2 (**B**), and p38 (**C**) phosphorylation. n = 4. (**D–F**) Effects of JNK inhibitor SP600125 (SP; 20 µM), ERK1/2 inhibitor PD98059 (PD; 20 µM) and p38 inhibitor SB203580 (SB; 10 µM) on IL-33 (100 ng/mL; 8 h)-induced NF-κB p65 phosphorylation. n = 3. Top panels are the representative immunoblots and bottom panels are the average data. Data are mean ± SEM. Statistical analysis was performed with one-way ANOVA test followed by the Newman-Keuls multiple comparison test. *p < 0.05, **p < 0.01 vs. 0 h or 0 ng/mL; #p < 0.05, ##p < 0.01, ###p < 0.001 vs. IL-33-treated group

IL-33 (100 ng/mL; 0, 0.5, 1, 2, 4 and 6 h) treatment increased the phosphorylation of JNK, ERK1/2 and p38, the three members of MAPK family, in a time-dependent manner with maximal effects observed at 2 h for JNK and at 1 h for both ERK1/2 and p38 in amnion fibroblasts (Fig. 5A-C). In addition, IL-33 (100 ng/mL; 8 h) also increased the phosphorylation of p65 (Fig. 5D-F), a subunit of NF-κB complex, which could be blocked by SP600125 (SP; 20 µM), PD98059 (PD; 20 µM) and SB203580 (SB; 10 µM), the respective inhibitors for JNK, ERK1/2 and p38.

**Fig. 6:**
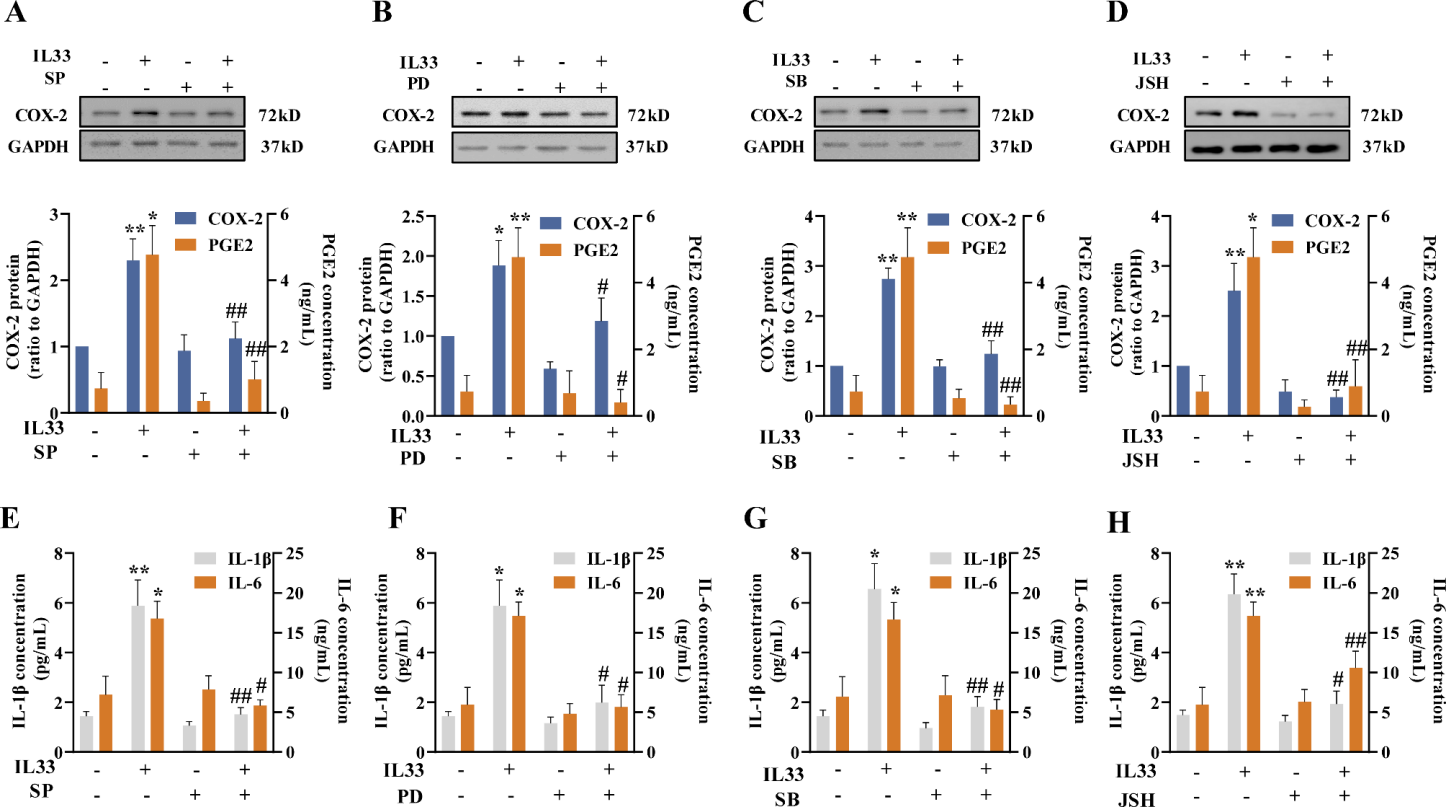
Involvements of JNK, ERK1/2, p38 and NF-κB in the induction COX-2, IL-1β and IL-6 by IL-33 in human amnion fibroblasts. (**A**–**D**) Blockade of IL-33 (100 ng/mL; 8 h)-induced COX-2 expression and PGE2 production by JNK inhibitor SP600125 (SP; 20 µM; A), ERK1/2 inhibitor PD98059 (PD; 20 µM; B), p38 inhibitor SB203580 (SB; 10 µM; C) and NF-κB inhibitor JSH-23 (JSH; 10 µM; D). n = 3. Top panels are the representative immunoblots and bottom panels are the average data. (**E–H**) Blockade of IL-33 (100 ng/mL; 8 h)-induced IL-1β and IL-6 production by JNK inhibitor SP600125 (SP; 20 µM; E), ERK1/2 inhibitor PD98059 (PD; 20 µM; F), p38 inhibitor SB203580 (SB; 10 µM; G) and NF-κB inhibitor JSH-23 (JSH; 10 µM; H). n = 4–5. Data are mean ± SEM. Statistical analysis was performed with one-way ANOVA test followed by the Newman-Keuls multiple comparison test. *p < 0.05, **p < 0.01 vs. 0 ng/mL; #p < 0.05, ##p < 0.01 vs. IL-33-treated group

Moreover, inhibition of JNK, ERK1/2, p38 and NF-κB with SP (20 µM), PD (20 µM), SB (10 µM) and JSH (10 µM) also blocked the induction of COX-2 abundance and secretion of PGE2, IL-1β and IL-6 in amnion fibroblasts by IL-33 (Fig. 6A-H). These results indicate that IL-33 can activate MAPKs and NF-κB in a sequential manner in human amnion fibroblasts to induce pro-inflammatory factors pertinent to parturition.

### Role of ST2 receptor in the effect of IL-33 in human amnion fibroblasts

**Fig. 7:**
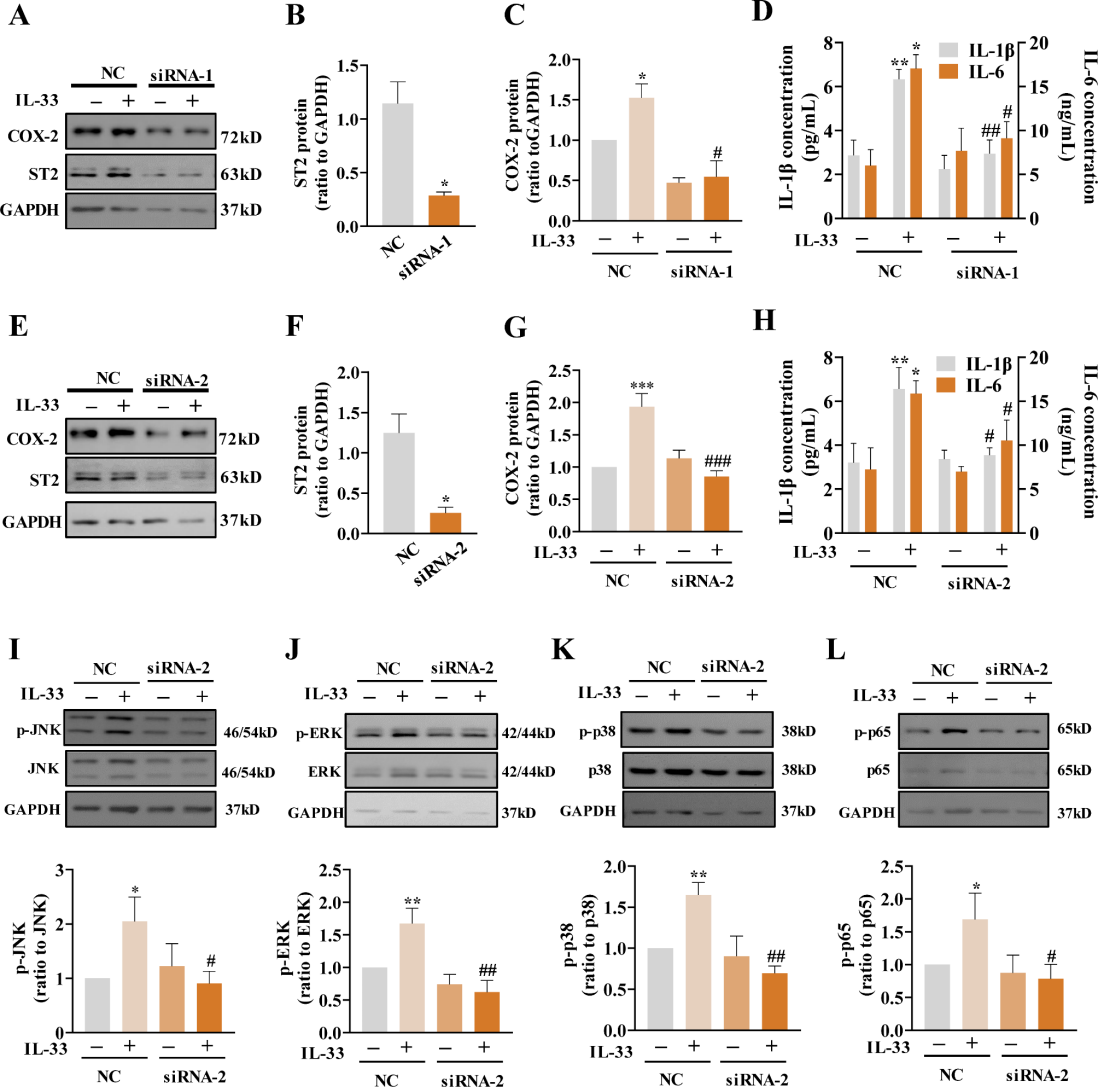
Role of ST2 in the induction of COX-2, IL-1β and IL-6 by IL-33 in human amnion fibroblasts. (**A-H**) Effects of siRNA-mediated knockdown of ST2 on the induction of COX-2, IL-1β and IL-6 by IL-33 (100 ng/mL; 8 h). A and E are the representative immunoblots. B and F are the knockdown efficiency of ST2 protein. n = 4–5. (**I-L**) Effects of siRNA-mediated knockdown of ST2 on the induction of the phosphorylation of JNK, ERK1/2, p38 and p65 by IL-33 (100 ng/mL; 1.5 h). n = 3–4. Top panels are the representative immunoblots and bottom panels are the average data. Data are mean ± SEM. Statistical analysis was performed with one-way ANOVA test followed by the Newman‐Keuls multiple comparison test. *p < 0.05, **p < 0.01, ***p < 0.001 vs. 0 ng/mL. #p < 0.05, ##p < 0.01, ###p < 0.001 vs. IL-33-treated groups

To investigate the role of ST2 receptor in the effect of IL-33 in human amnion fibroblasts, siRNA-mediated knock-down of ST2 was conducted. The efficiency of ST2 knockdown was shown in Fig. 7B and F. We found that siRNA-mediated knockdown of ST2 blocked not only the induction of COX-2, IL-1β, IL-6 (Fig. 7A-H) but also the induction of phosphorylation of JNK, ERK1/2, p38 MAPKs and p65 (Fig. 7I-L) by IL-33 (100 ng/mL) in amnion fibroblasts. These results suggest that these effects of IL-33 are mediated by the ST2 receptor in human amnion fibroblasts.

### Induction of preterm birth by IL-33 in pregnant mice

**Fig. 8:**
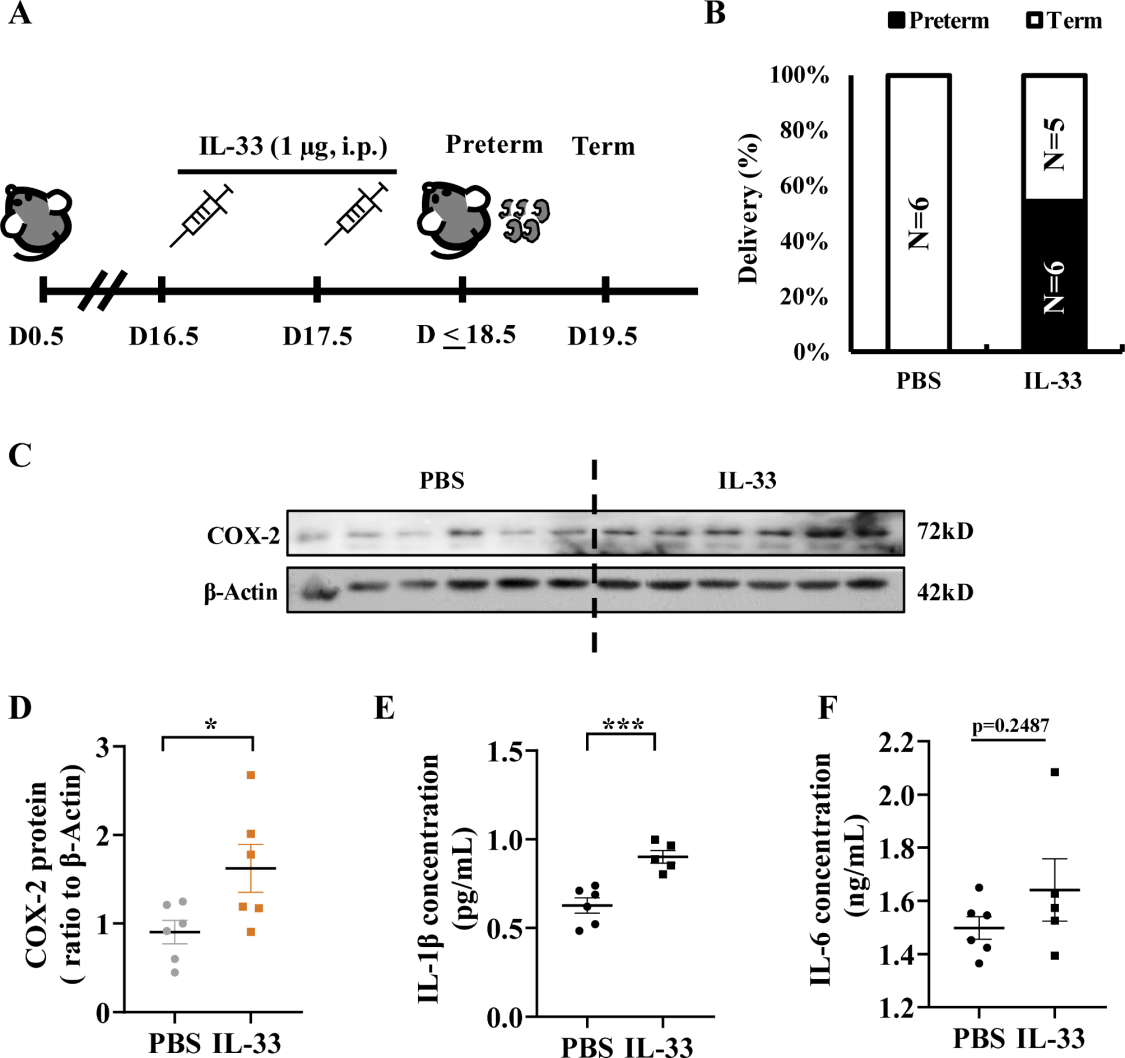
Induction of preterm birth by IL-33 in pregnant mice. (**A**) Time-line illustrating the procedure of IL-33 administration in pregnant mice. i.p., intraperitoneal injection. (**B**) Preterm birth rate in IL-33 administration (n = 11) and vehicle control (n = 6) group. (**C and D**) COX-2 protein abundance in the fetal membranes collected from IL-33 (n = 6) and vehicle control (n = 6) groups. C is the immunoblot, D is the average data. (**E and F**) IL-1β and IL-6 abundance in the fetal membranes collected from IL-33 (n = 5) and vehicle control (n = 6) groups. Data are mean ± SEM. Statistical analysis was performed with unpaired Student’s t-test. *p < 0.05, ***p < 0.001 vs. vehicle control

Intraperitoneal injection of IL-33 (1 µg per dam; once a day) on 16.5 and 17.5 dpc (Fig. 8A) led to a 55% preterm birth rate in mice (Fig. 8B), along with significantly increased COX-2 and IL-1β abundance in the fetal membranes (Fig. 8C-E). The abundance of IL-6 also showed a trend of increase but did not reach statistical significance (Fig. 8F).

## Discussion

In this study, we have provided evidence that IL-33/ST2 axis is present in human amnion fibroblasts, and its activation may be involved in the inflammatory reaction of the amnion by increasing the production of inflammatory factors pertinent to parturition in both term and preterm labor. We found that IL-1β, IL-6 and PGE2 were among inflammatory factors which were induced by IL-33 via activation of the MAPKs-NF-κB pathway in human amnion fibroblasts. Since the expression of IL-33 can be induced by LPS, SAA1 and IL-1β, the crucial molecules involved in both infectious and non-infectious inflammation of the fetal membranes (Keelan et al. 2003; Li et al. 2017; Ye et al. 2015), we believe that our findings may carry significance in both normal term labor and spontaneous preterm labor. Notably, we found that IL-33 also induced its own production in amnion fibroblasts, indicating that there may a feed-forward expression of IL-33 in amnion fibroblasts in parturition. The important role of IL-33 in parturition was endorsed by our findings that IL-33 administration could indeed induce preterm birth in the mouse.

Previous studies have demonstrated that IL-33 plays an essential role in several pregnancy-related diseases, including endometriosis, recurrent pregnancy loss and preeclampsia, etc.(Chen H. et al. 2018; Fock et al. 2013; Granne et al. 2011; Miller J. E. et al. 2017; Salker et al. 2012; Zhao et al. 2021). Unlike most of other cytokines, IL-33 can function either as a secreted cytokine or as an intracellular nuclear factor regulating gene expression (Bertheloot et al. 2017; Cayrol et al. 2018; 2022). Interestingly, Chen et al. found that the abundance of IL-33 in the nucleus of the myometrial cell decreases with the onset of labor, and they postulated that the reduction of nuclear IL-33 may help the transition of myometrium from a quiescent state to a contractile state at parturition (Chen et al. 2022). In this study, we demonstrated that secreted IL-33 played an opposite role in the onset of labor in the amnion, which may explain why there are contradictory changes in the nuclear and secreted IL-33 abundance at parturition though we are unclear about the concurrent changes of nuclear IL-33 abundance in amnion fibroblasts at the current stage. We speculate that there may a switch of nuclear IL-33 to IL-33 secretion at parturition so that IL-33 can function as a cytokine to exert its parturition-initiating effects through the ST2 receptor in the amnion.

In this study, we found that the effect of IL-33 on inflammatory factors was mediated by ST2 in human amnion fibroblasts. ST2 is present in two main differentially spliced variants: a membrane-bound form (ST2 or ST2L) mediating the cytokine effect of IL-33, and a soluble form (sST2), which is also known as a decoy receptor inhibiting the effect of IL-33 (Griesenauer et al. 2017). In this study, we found that the protein abundance of ST2 increased in the amnion at parturition, while sST2 protein was undetectable, indicating that ST2 but not sST2 is predominantly expressed in human amnion at parturition. The important role of ST2 in mediating the effect of IL-33 was illustrated by siRNA-mediated knockdown of ST2 expression in amnion fibroblasts, which substantially abolished the effects of IL-33.

Activation of the IL-33/ST2 axis has been linked to the activation of MAPKs and NF-κB pathways in a variety of cell types including dendritic cells, mast cells, osteoblastic cells, etc.(Helbig et al. 2020; Huang et al. 2021; Huang S. J. et al. 2018; McCarthy et al. 2019; Mine et al. 2014; Pinto et al. 2018). Here in this study, we also demonstrated that MAPKs and NF-κB pathways underpinned the actions of IL-33 in human amnion fibroblasts. In mammals, MAPKs can be grouped into three main subfamilies, namely, ERK, p38, and JNK, which have been demonstrated to be associated with both infection and non-infection-induced inflammatory responses (Arthur et al. 2013; Li et al. 2017; Ni et al. 2022; Rincón et al. 2009; Rodriguez-Barbero et al. 2006). In this study, we found that all three MAPKs were involved in the induction of IL-1β, IL-6 and PGE2 production by IL-33 in amnion fibroblasts, further indicating a crucial role of MAPKs in the inflammatory responses of the amnion at parturition. NF-κB is a key inflammatory transcription factor (Liu et al. 2017), and the transcriptional activity of NF-κB can be activated following the activation of MAPK signaling cascades (Li et al. 2017; Schulze-Osthoff et al. 1997). Here, we also demonstrated that NF-κB could be activated following the activation of MAPKs in human amnion fibroblasts. Given the classical role of NF-κB in the mediation of the expression of a wide array of pro-inflammatory mediators in inflammation, we speculate that there may be other IL-33-inducible pro-inflammatory mediators in addition to IL-1β, IL-6 and COX-2 in amnion fibroblasts in parturition. Undoubtedly, it should be an interesting issue to explore with in the future.

## Conclusions

**Fig. 9:**
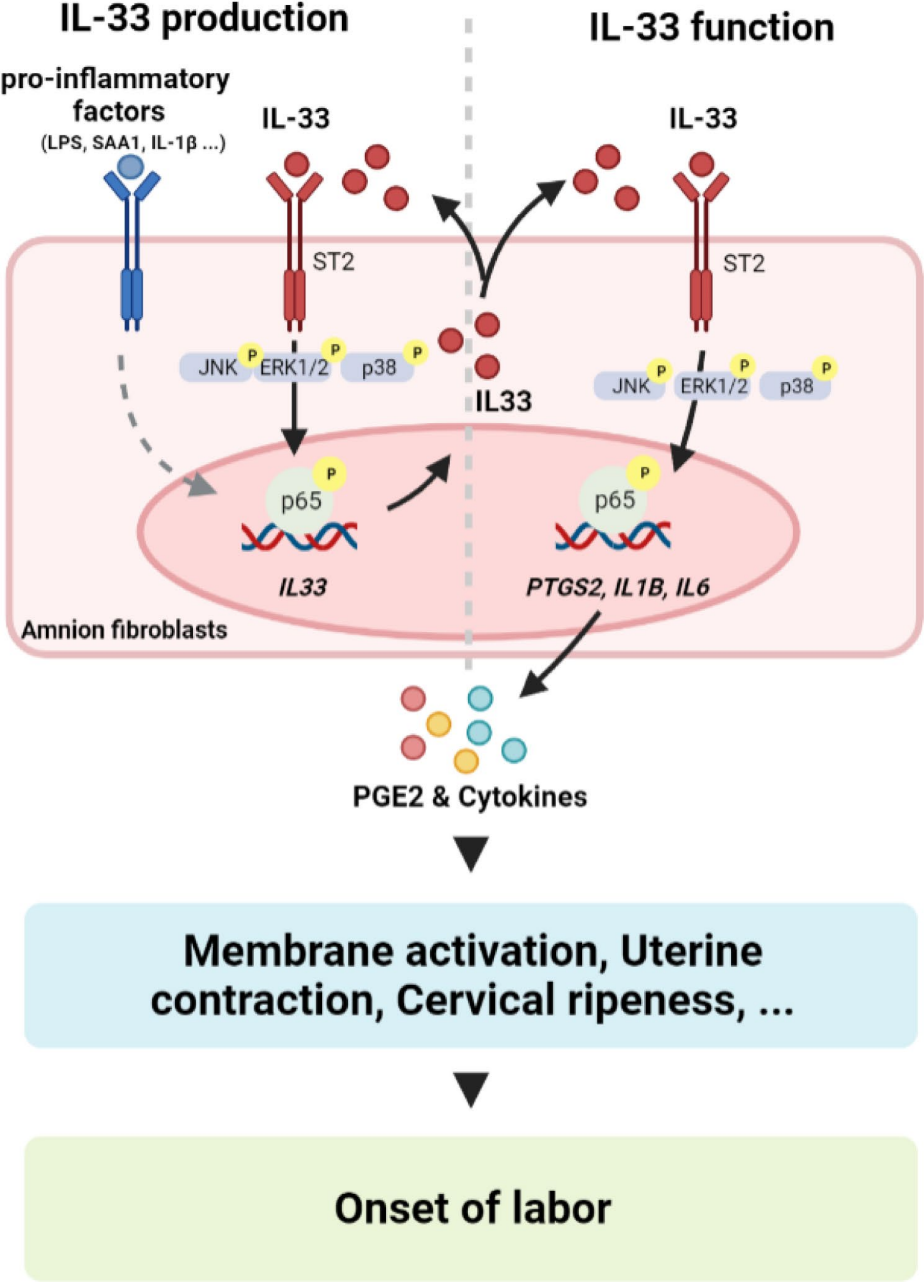
Diagram illustrating the involvement of IL-33/ST2 axis in inflammatory reactions in human amnion fibroblasts. The inflammatory mediators pertinent to parturition such as LPS, IL-1β and SAA1 stimulate the expression of IL-33 expression and secretion via activation of NF-κB. In turn, via binding to ST2 receptor, IL-33 activates JNK, ERK1/2 and p38 by phosphorylation, which subsequently leads to the activation of NF-κB through phosphorylation of its subunit p65 resulting in increased expression of inflammatory factors including IL-1β, IL-6 as well as COX-2, the rate-limiting enzyme in PGE2 synthesis. The increased production of PGE2 and pro-inflammatory cytokines participate in fetal membrane activation, uterine contraction and cervical ripening at parturition

In conclusion, we have demonstrated in this study that there is activation of the IL-33/ST2 axis in human amnion fibroblasts in term and preterm labor, which is associated with inflammatory responses of the amnion. Activation of the IL-33/ST2 axis leads to increased production of inflammatory factors pertinent to parturition (Fig. 9). Targeting the IL-33/ST2 axis may have potential value in the treatment of preterm birth.

## Electronic supplementary material

Below is the link to the electronic supplementary material.

**Supplementary Figure S1**. Abundance of GAPDH and β-Actin in the human amnion. Western blotting revealed that the abundance of GAPDH and β-Actin was constant across amnion tissue samples collected from TL (Term labor, n=7) and TNL (Term non-labor, n=7) patients. **Supplementary Figure S2**. Abundance of IL-33 and ST2 in the human amnion at term and preterm labor. Abundance of IL-33 and ST2 protein in the amnion of TL (term labor, n=6) and PL (preterm labor, n=7) groups as measured with Western blotting. Top panels are the immunoblots. ns, non-significant. Data are mean ± SEM. Statistical analysis was performed with the Mann?Whitney U test. **Supplementary Figure S3**. Distribution of IL-33 and ST2 in the human amnion obtained from patients with preterm labor. (A and B) Immunohistochemical staining of IL-33 (A) and ST2 (B) in the human amnion showing the presence of IL-33 and ST2 in both epithelial cells and fibroblasts. AE, amnion epithelial cells; AF, amnion fibroblasts. **Table S1** Primer sequences used for qRT-PCR.

Supplementary Material 2

## Data Availability

The transcriptomic sequencing data of the amnion tissue obtained from TL and TNL have been submitted to the GEO data repository (GSE166453). The original data and materials presented in the study are available from the corresponding authors upon reasonable request.

## List of abbreviations

IL-33: Interleukin-33
ST2: Suppression of tumorigenicity 2
PGs: Prostaglandins
NF-κB: Nuclear factor-kappa B
MAPK: Mitogen-activated protein kinase
LPS: Lipopolysaccharide
SAA1: Acute-phase protein serum amyloid A1
COX-2: Cyclooxygenase-2
NCBI: National Center for Biotechnology Information
FPKM: Fragments per kilo base per million mapped reads
GEO: Gene Expression Omnibus
qRT-PCR: Quantitative real-time polymerase chain reaction
TNL: Term non-labor
TL: Term labor
PNL: preterm non-labor
PL: Preterm labor
PBS: Phosphate buffer saline
RIPA: Radioimmunoprecipitation assay
DMEM: Dulbecco’s Modified Eagle Medium
FBS: Fetal bovine serum
siRNA: Small interfering RNA
GAPDH: Glyceraldehyde 3-phosphate dehydrogenase
