# Supplementary material for: IL-33/ST2 axis of human amnion fibroblasts participates in inflammatory reactions at parturition
Source: Mol Med. 2023 Jul 4;29:88. doi: 10.1186/s10020-023-00668-9 (PMC10318762; doi:10.1186/s10020-023-00668-9)
Supplement: Supplementary file 1 — Supplementary Figure S1. Abundance of GAPDH and β-Actin in the human amnion. Western blotting revealed that the abundance of GAPDH and β-Actin was constant across amnion tissue samples collected from TL (Term labor, n=7) and TNL (Term non-labor, n=7) patients. Supplementary Figure S2. Abundance of IL-33 and ST2 in the human amnion at term and preterm labor. Abundance of IL-33 and ST2 protein in the amnion of TL (term labor, n=6) and PL (preterm labor, n=7) groups as measured with Western blotting. Top panels are the immunoblots. ns, non-significant. Data are mean ± SEM. Statistical analysis was performed with the Mann?Whitney U test. Supplementary Figure S3. Distribution of IL-33 and ST2 in the human amnion obtained from patients with preterm labor. (A and B) Immunohistochemical staining of IL-33 (A) and ST2 (B) in the human amnion showing the presence of IL-33 and ST2 in both epithelial cells and fibroblasts. AE, amnion epithelial cells; AF, amnion fibroblasts. Table S1 Primer sequences used for qRT-PCR. [file 10020_2023_668_MOESM1_ESM.pdf]

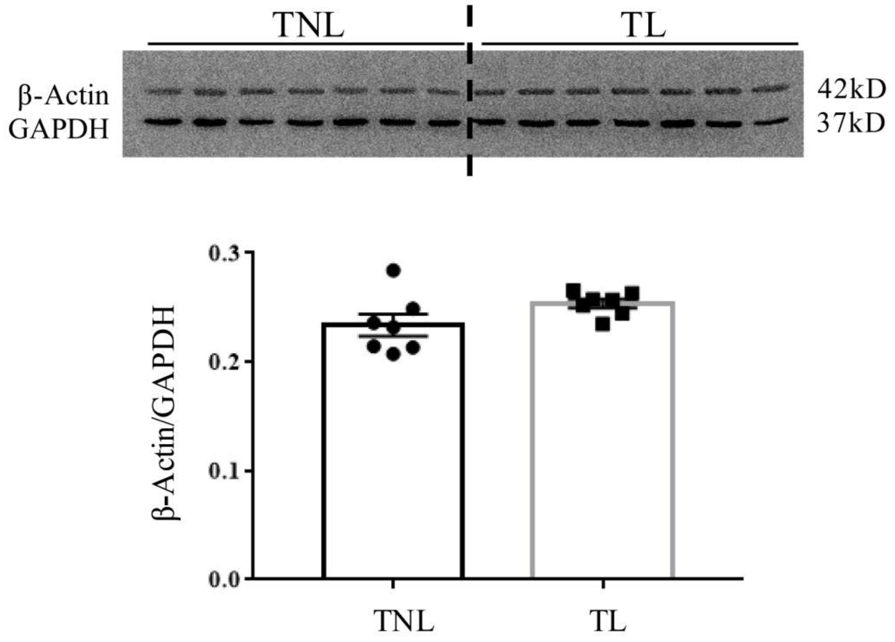

Supplementary Figure S1. Abundance of GAPDH and β-Actin in the human amnion. Western blotting revealed that the abundance of GAPDH and β-Actin was constant across amnion tissue samples collected from TL (Term labor, n=7) and TNL (Term non-labor, n=7) patients.

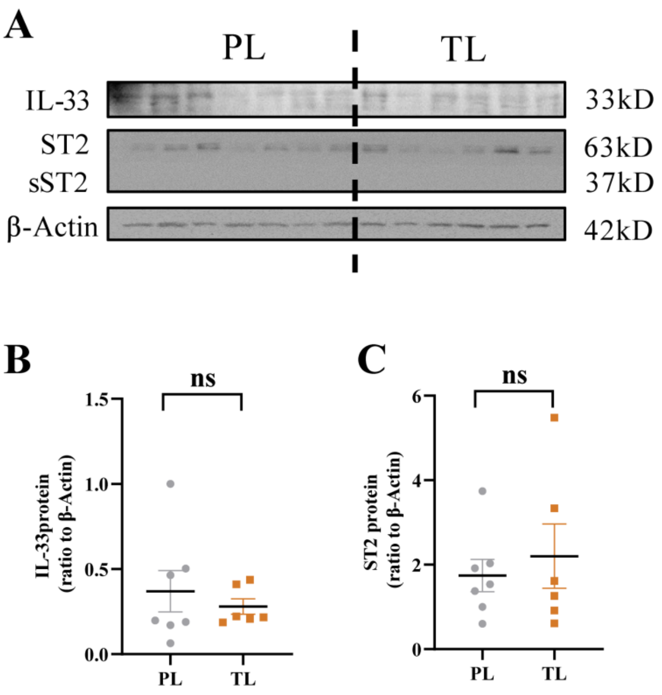

Supplementary Figure S2. Abundance of IL-33 and ST2 in the human amnion at term and preterm labor. Abundance of IL-33 and ST2 protein in the amnion of TL (term labor, n=6) and PL (preterm labor, n=7) groups as measured with Western blotting. Top panels are the immunoblots. ns, non-significant. Data are mean ± SEM. Statistical analysis was performed with the Mann–Whitney U test.

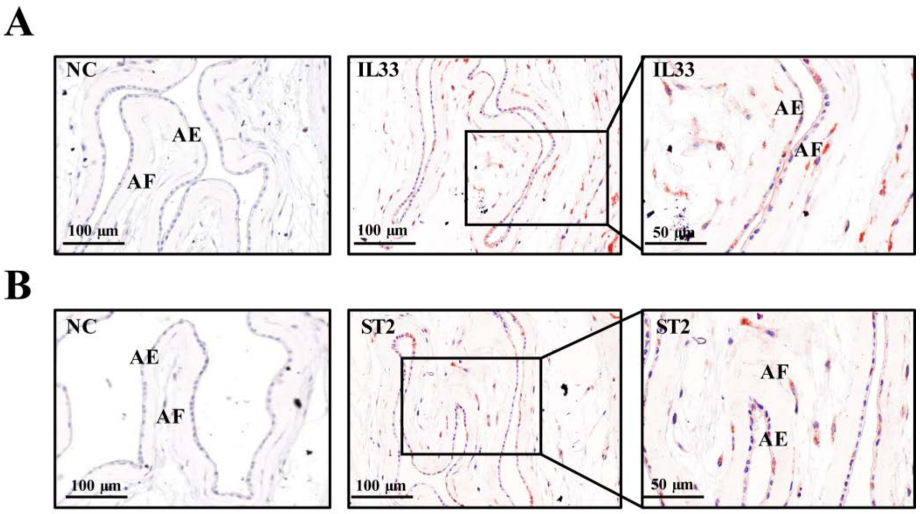

Supplementary Figure S3. Distribution of IL-33 and ST2 in the human amnion obtained from patients with preterm labor. (A and B) Immunohistochemical staining of IL-33 (A) and ST2 (B) in the human amnion showing the presence of IL-33 and ST2 in both epithelial cells and fibroblasts. AE, amnion epithelial cells; AF, amnion fibroblasts.

Table S1 Primer sequences used for qRT-PCR.

Genes	Primer sequences
IL-33	Forward: 5′-GCCTGTCAACAGCAGTCTACTG-3’
	Reverse: 5′-TGTGCTTAGAGAAGCAAGATACTC-3’
IL1RL1	Forward: 5′- CTCTGTTTCCAGTAATCGGAGCC-3’
	Reverse: 5′- GCAGCCAAGAACTGAGTGCCTT-3’
PTGS2	Forward: 5′-TGTGCAACACTTGAGTGGCT-3’
	Reverse: 5′- ACTTTCTGTACTGCGGGTG-3’
IL1B	Forward: 5′-CCACAGACCTTCCAGGAGAATG-3’
	Reverse: 5′-GTGCAGTTCAGTGATCGTACAGG-3’
IL6	Forward: 5′-AGACAGCCACTCACCTCTTCAG-3’
	Reverse: 5′- TTCTGCCAGTGCCTCTTTGCTG-3’
GAPDH	Forward: 5′- CCCCTCTGCTGATGCCCCCA -3’
	Reverse: 5′- TGACCTTGGCCAGGGGTGCT-3’
