# Supplementary material for: IL-33/ST2 axis of human amnion fibroblasts participates in inflammatory reactions at parturition
Source: Mol Med. 2023 Jul 4;29:88. doi: 10.1186/s10020-023-00668-9 (PMC10318762; doi:10.1186/s10020-023-00668-9)

Full unedited gel for Figure 1G

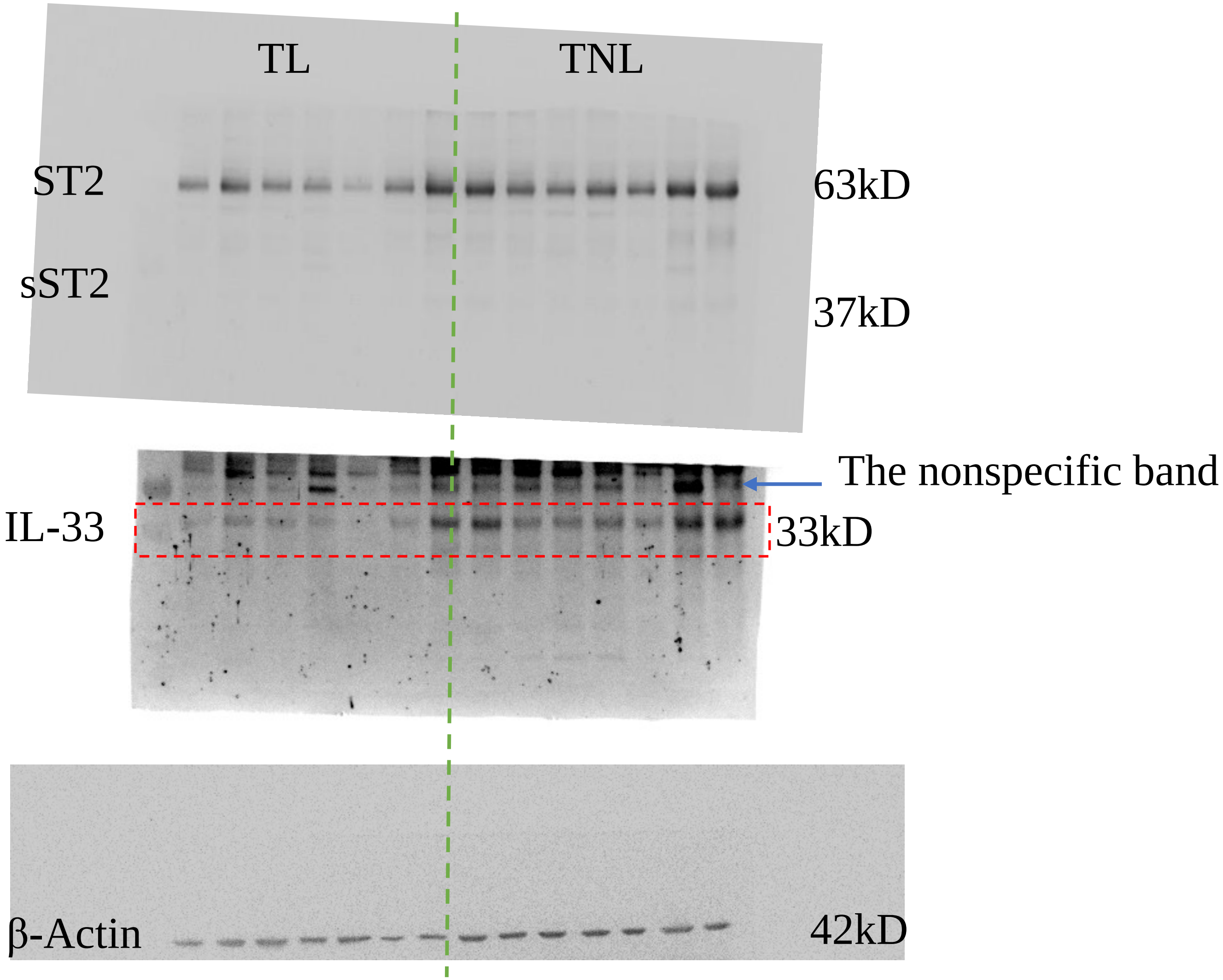

Full unedited gel for Figure 1H

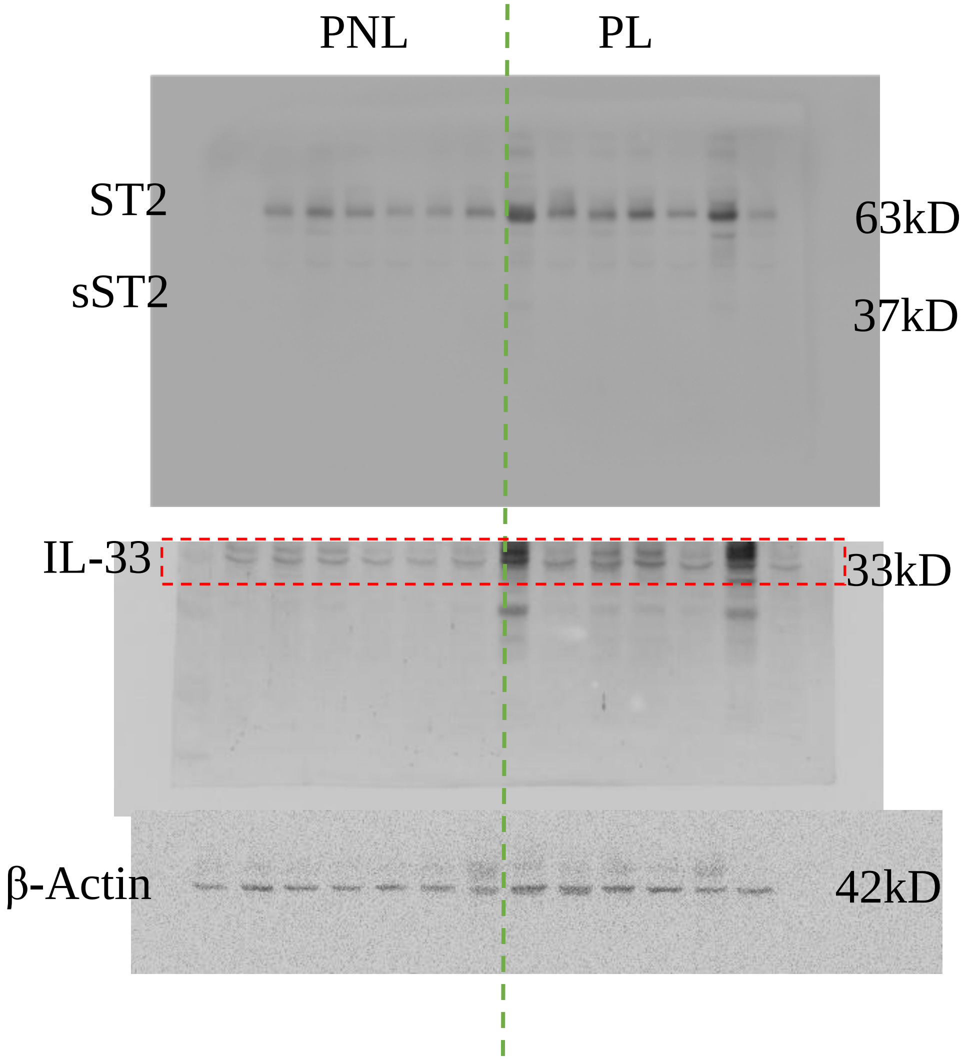

Full unedited gel for Figure 2E

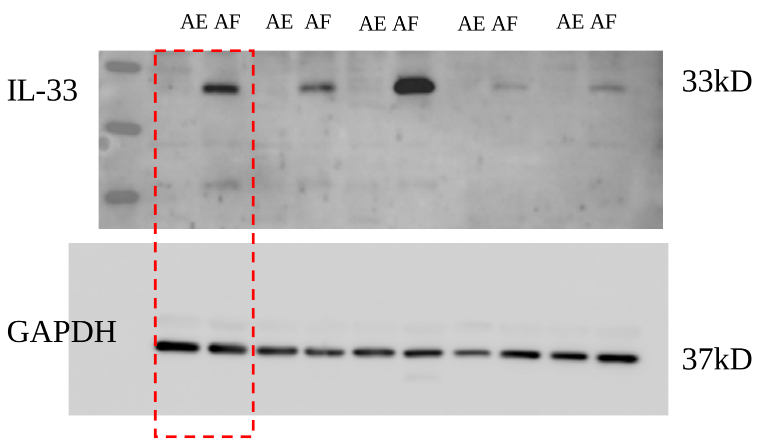

Full unedited gel for Figure 2F

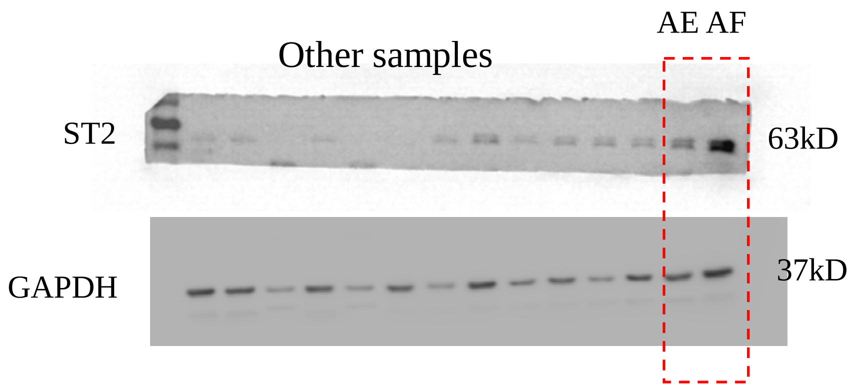

Full unedited gel for Figure 3A

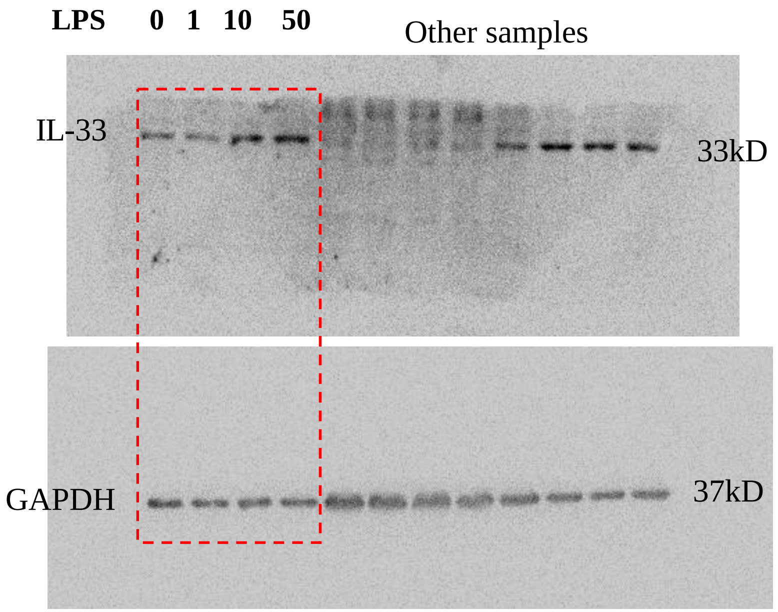

Full unedited gel for Figure 3B

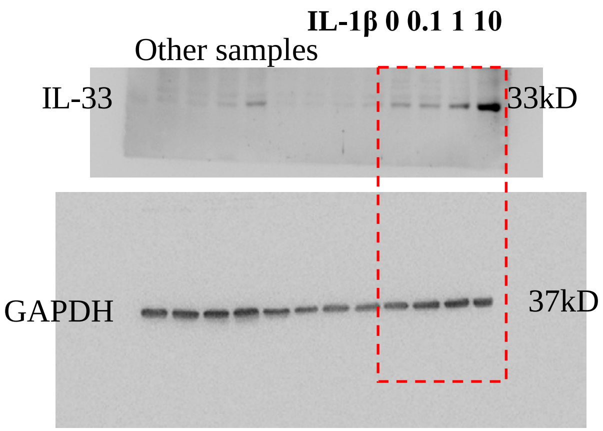

Full unedited gel for Figure 3C

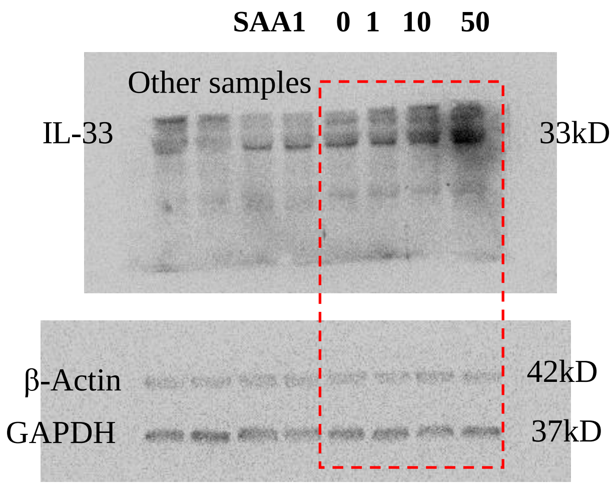

Full unedited gel for Figure 3D

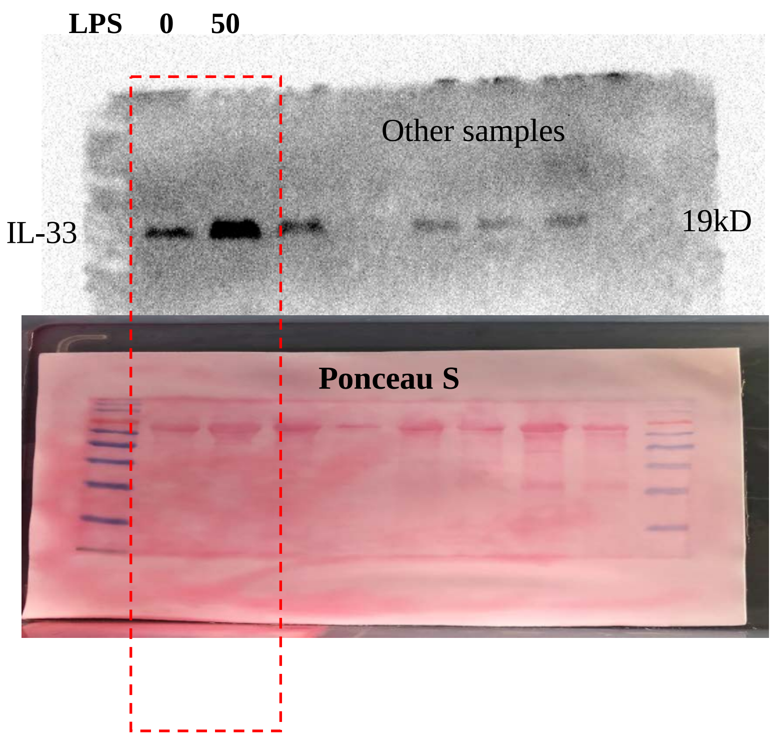

Full unedited gel for Figure 3E

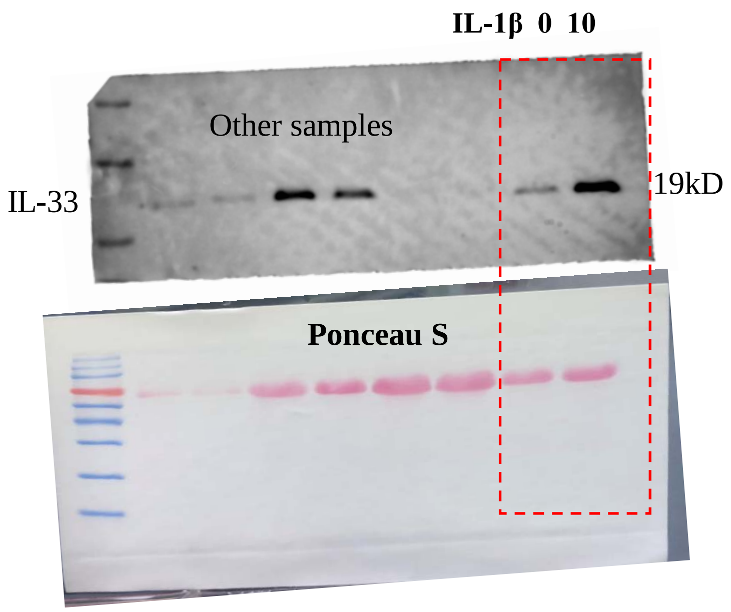

Full unedited gel for Figure 3F

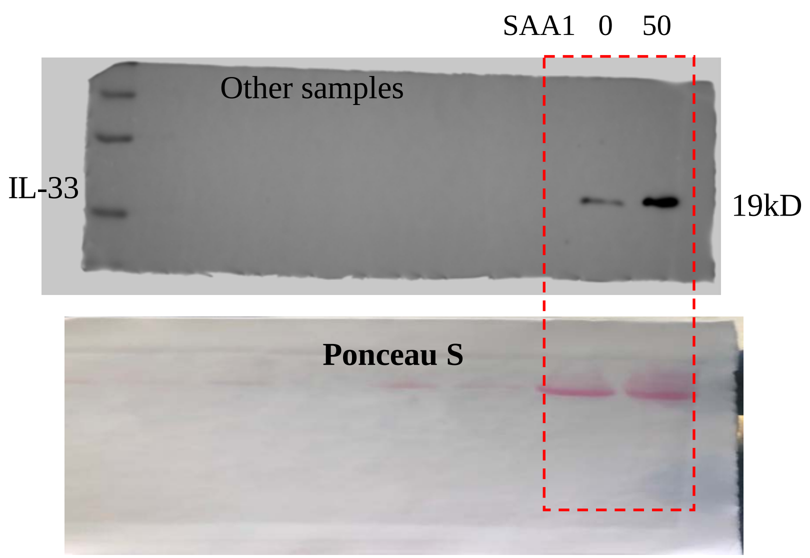

Full unedited gel for Figure 3G

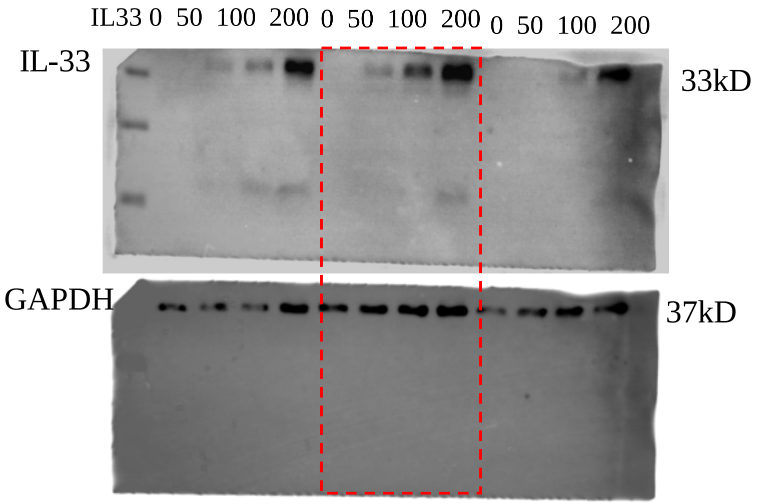

Full unedited gel for Figure 3H

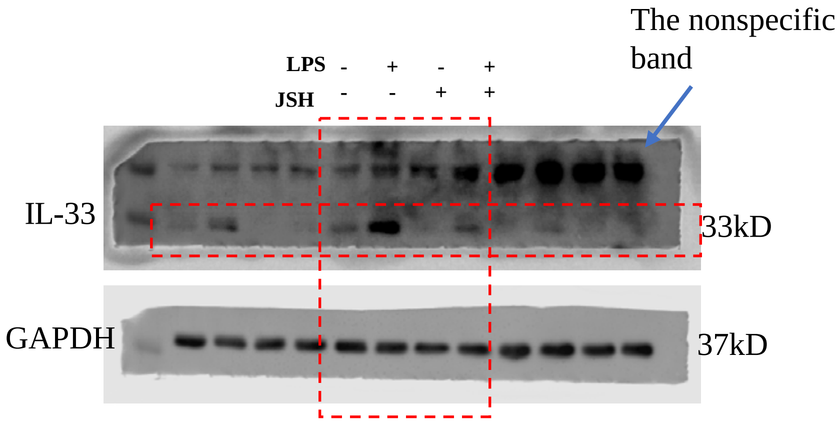

Full unedited gel for Figure 3I

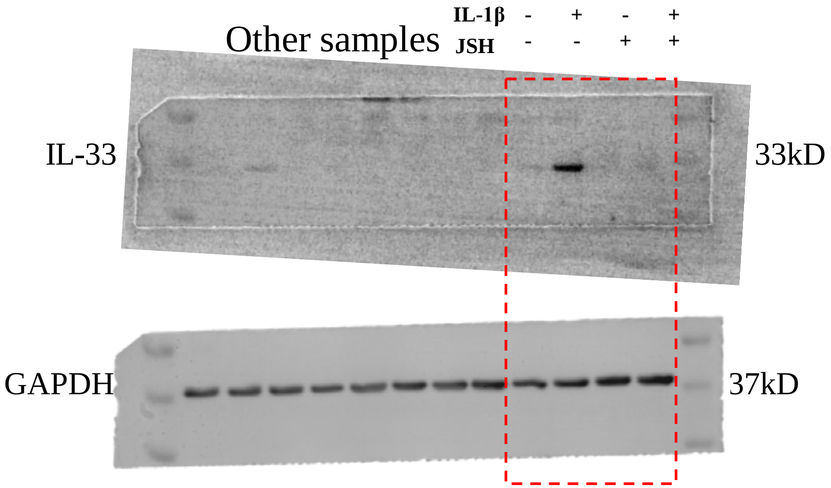

Full unedited gel for Figure 3J

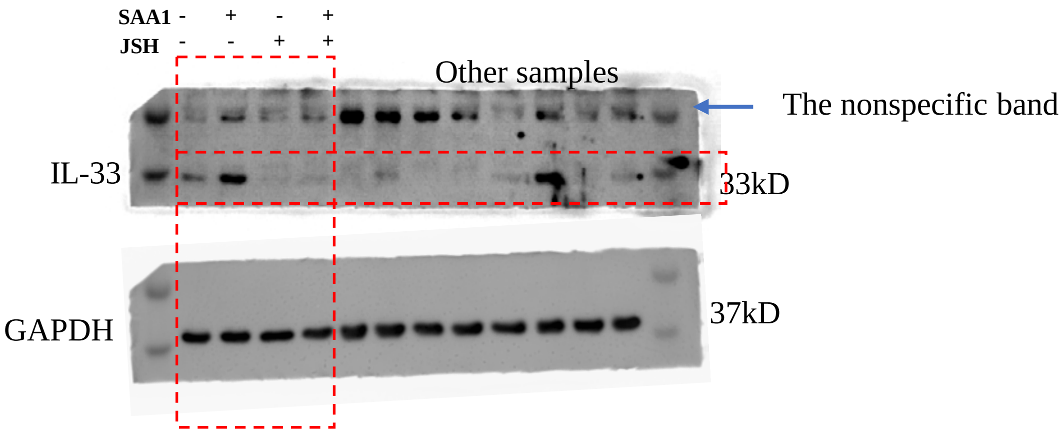

Full unedited gel for Figure 3K

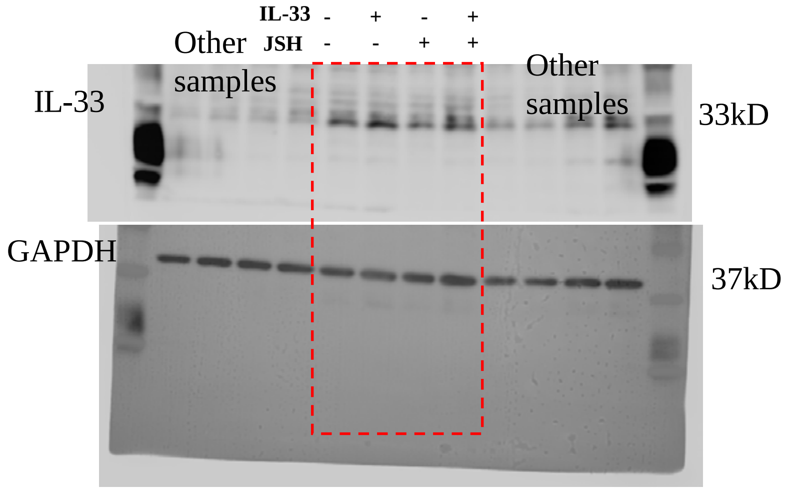

Full unedited gel for Figure 4G

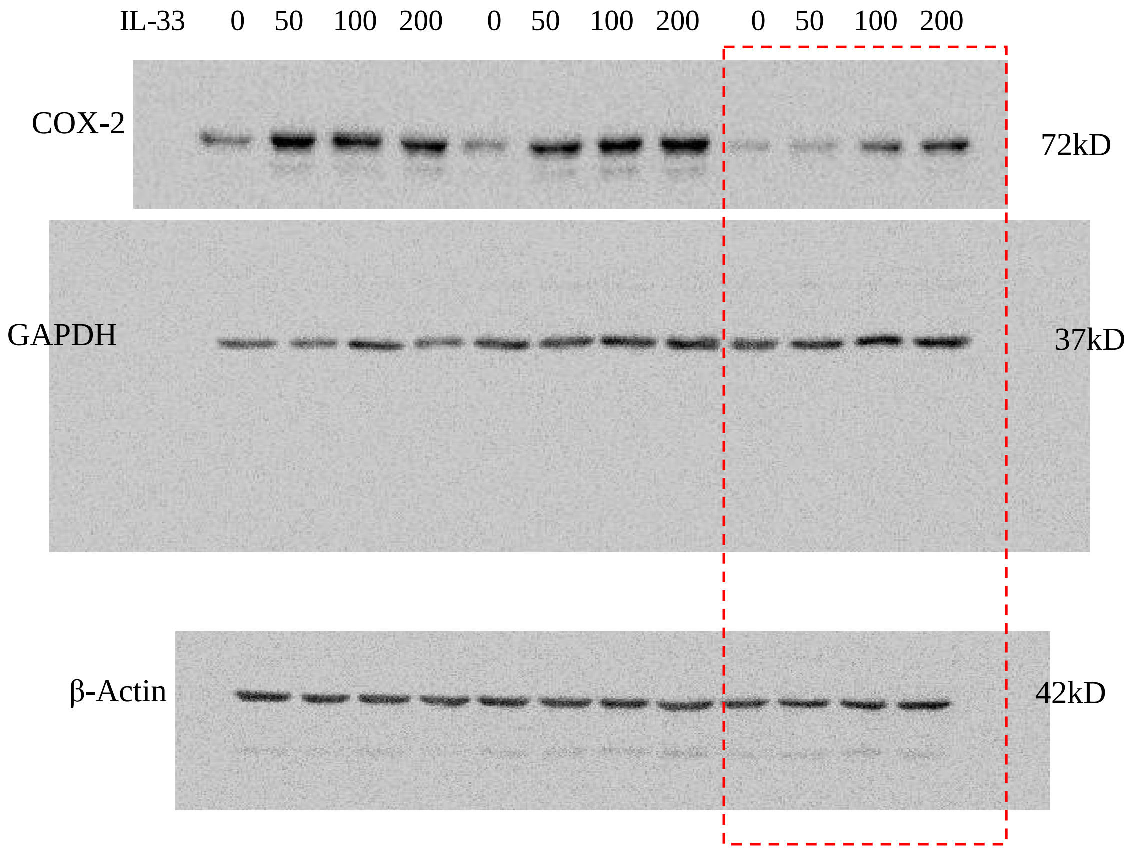

Full unedited gel for Figure 5A

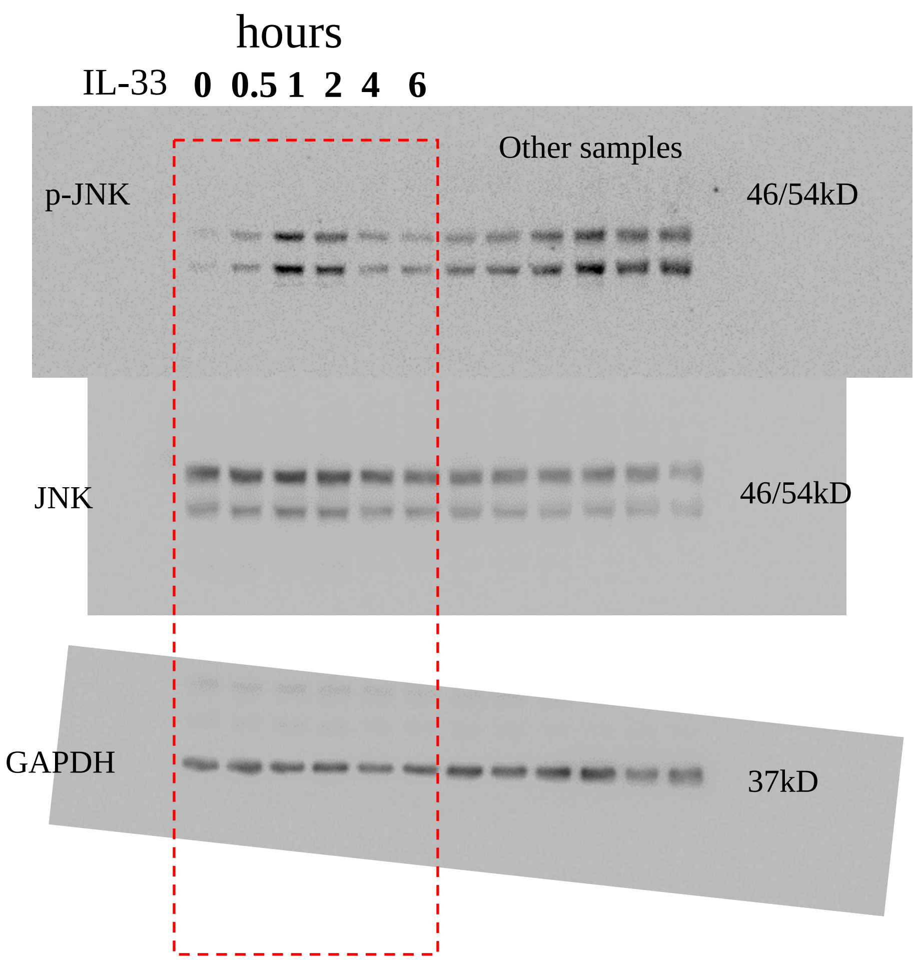

Full unedited gel for Figure 5B

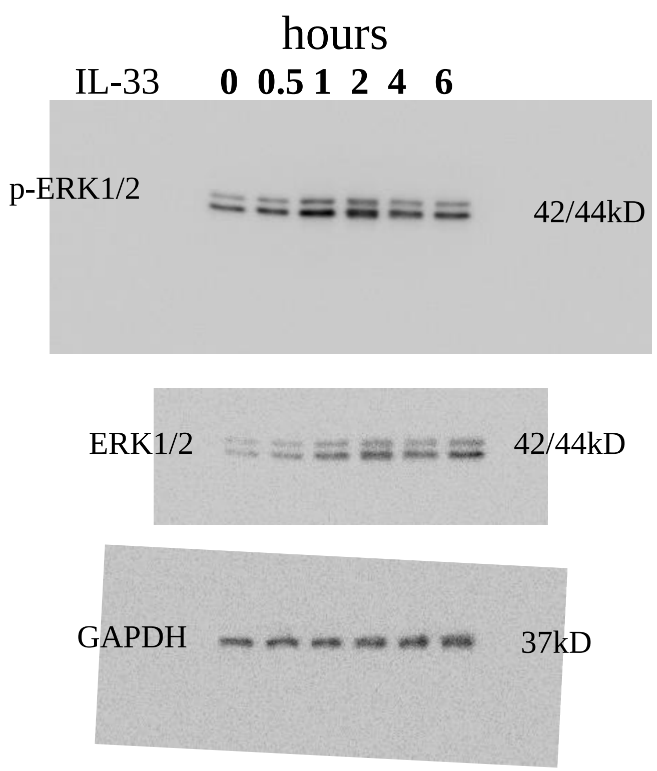

Full unedited gel for Figure 5C

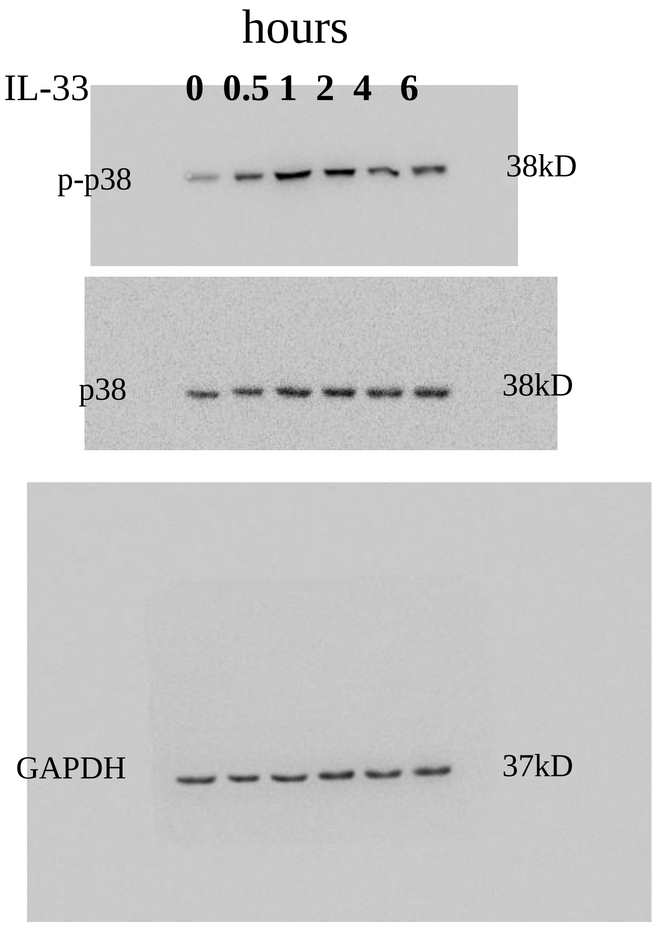

Full unedited gel for Figure 5D

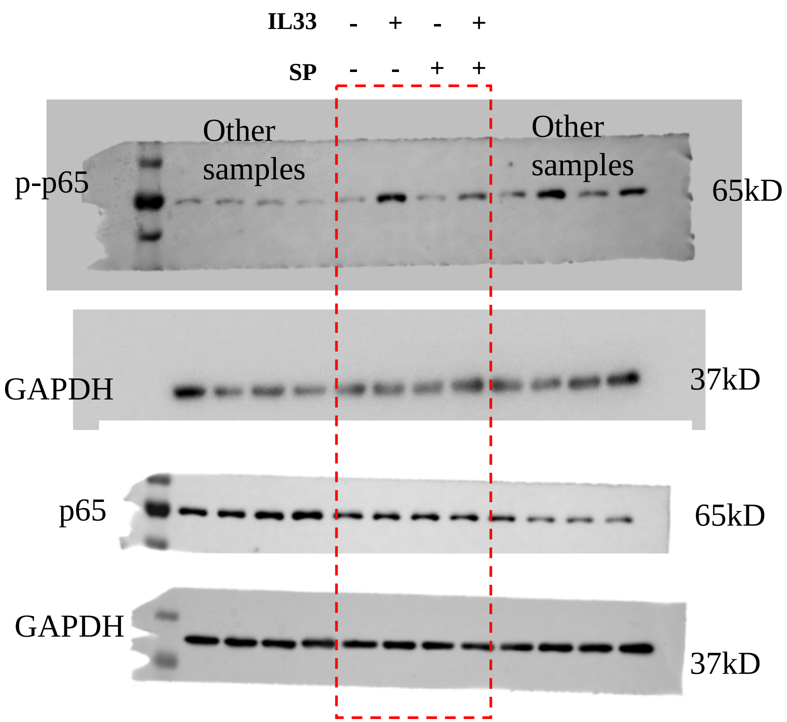

Full unedited gel for Figure 5E

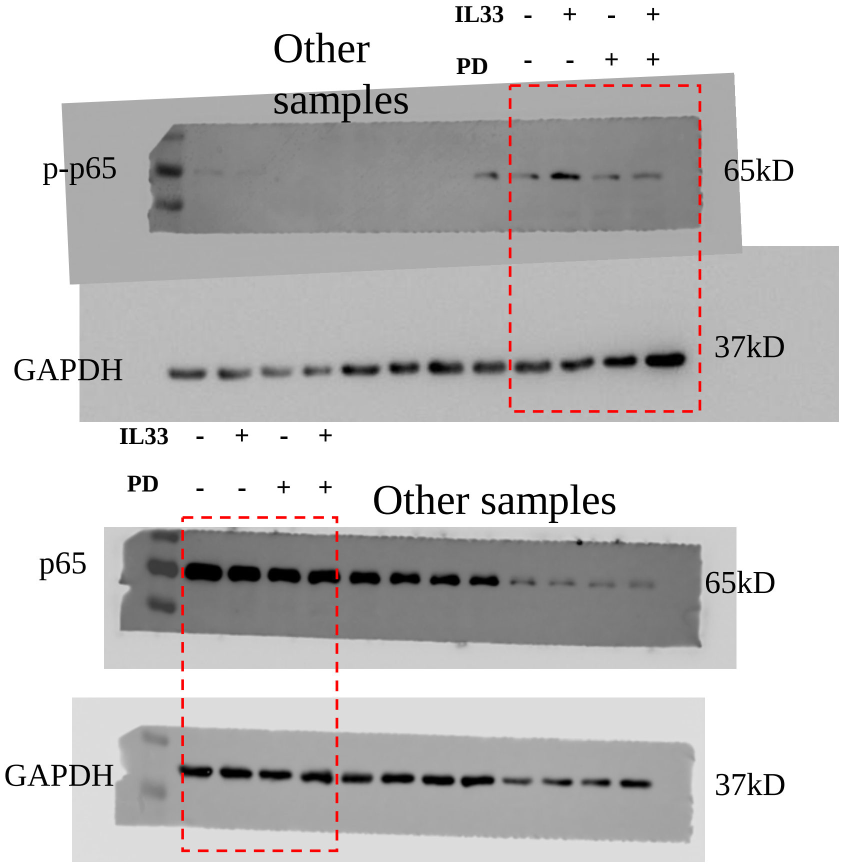

Full unedited gel for Figure 5F

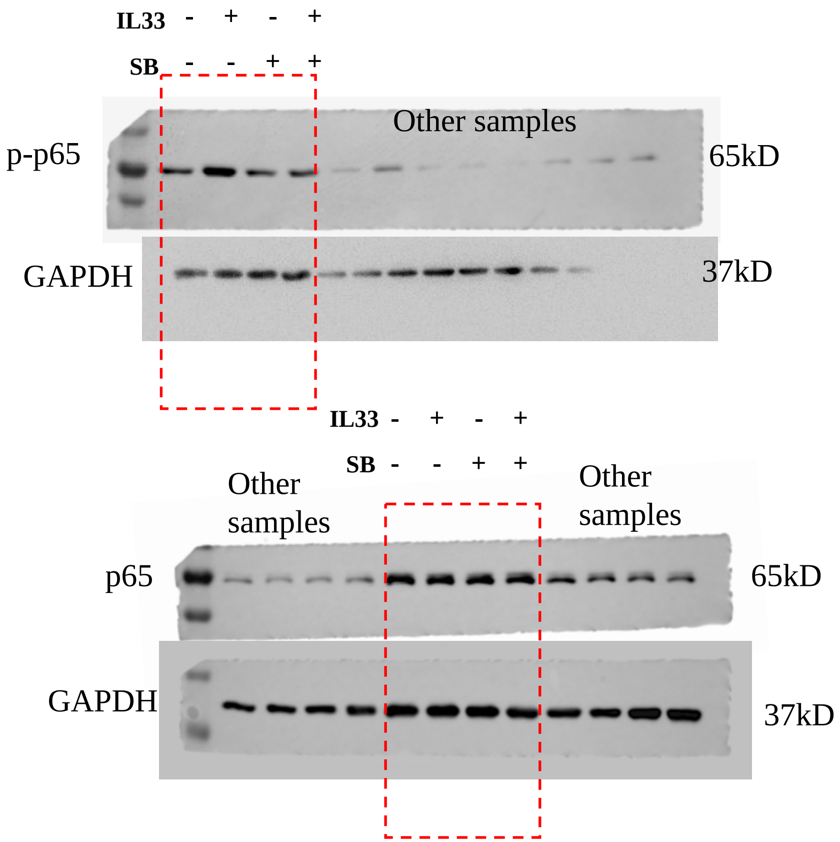

Full unedited gel for Figure 6A

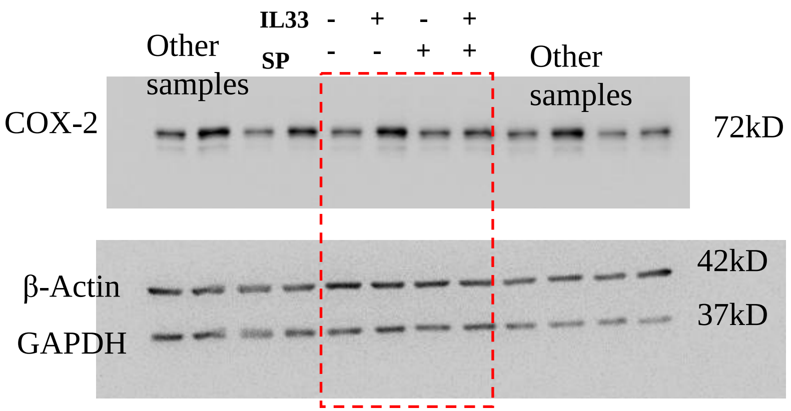

Full unedited gel for Figure 6B

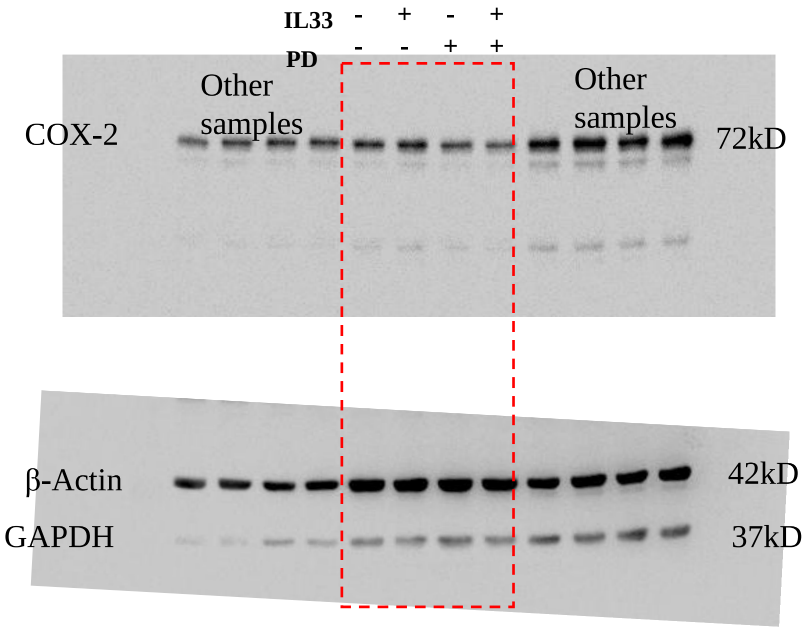

Full unedited gel for Figure 6C

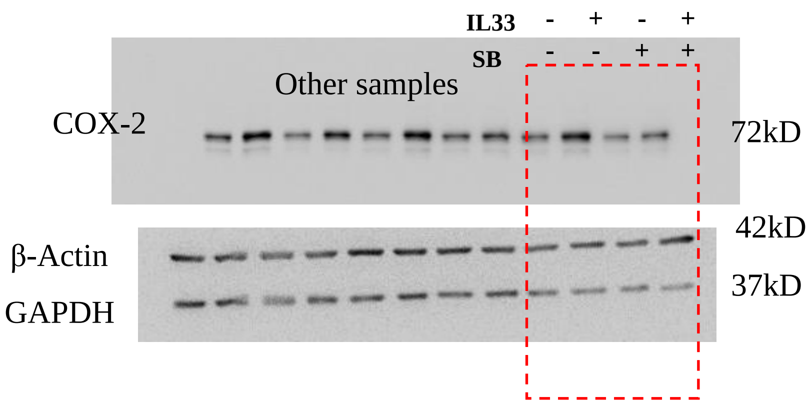

Full unedited gel for Figure 6D

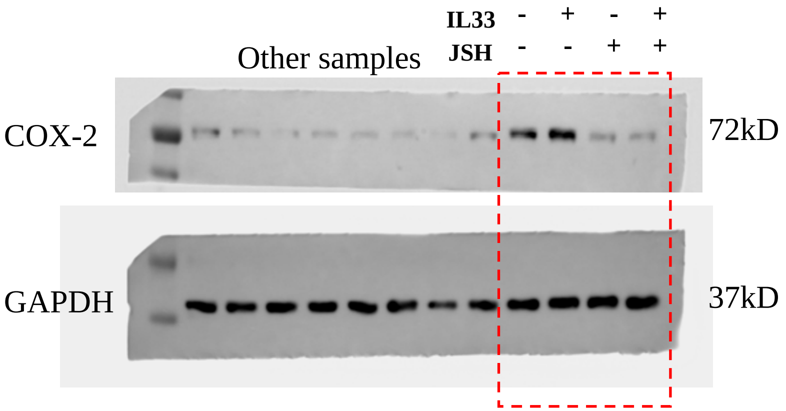

Full unedited gel for Figure 7A

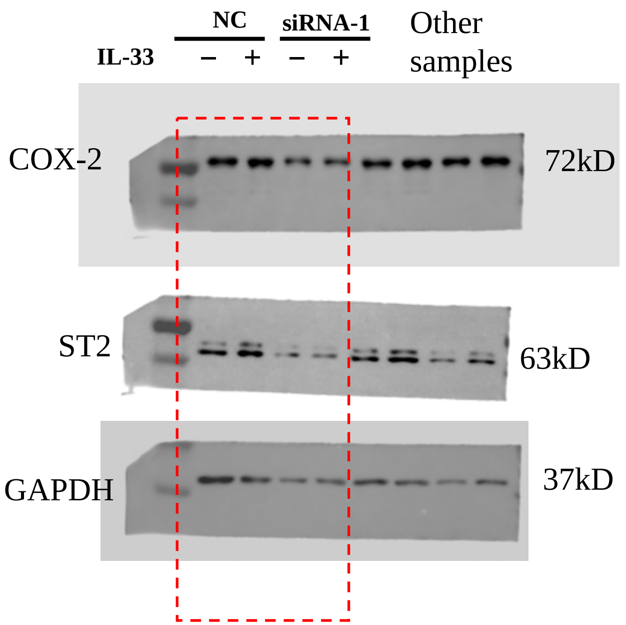

Full unedited gel for Figure 7E

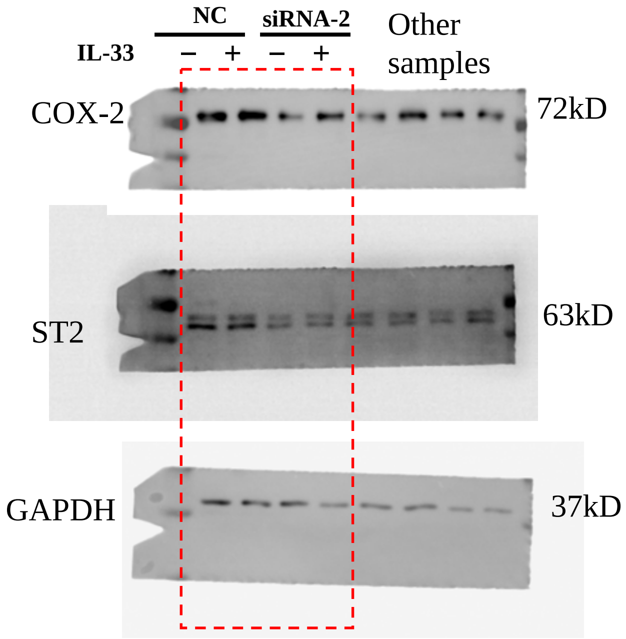

Full unedited gel for Figure 7I

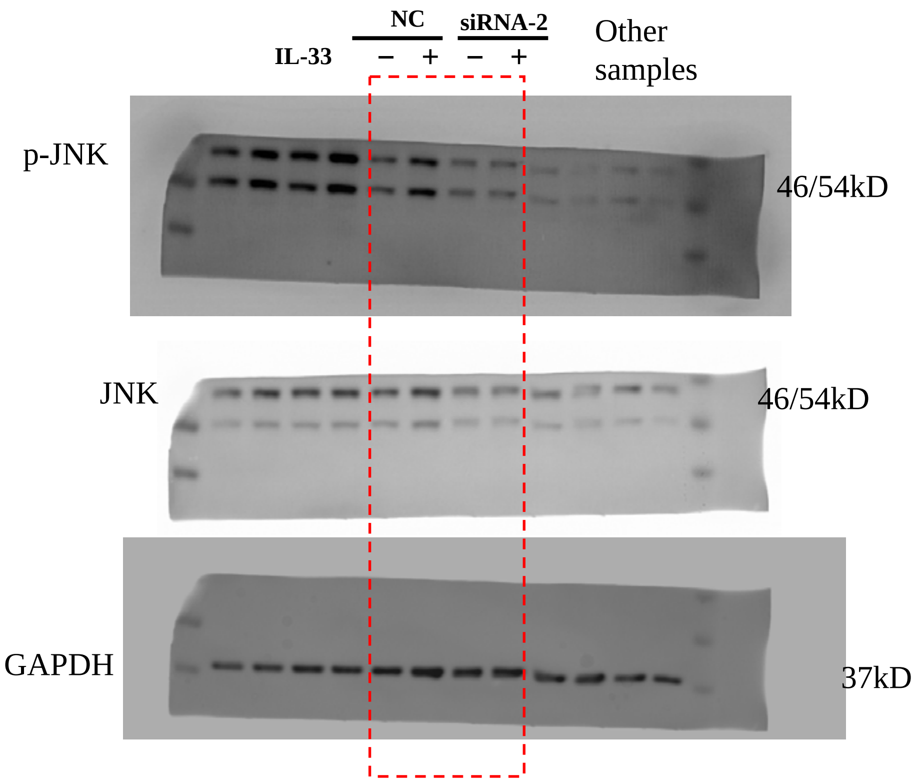

Full unedited gel for Figure 7J

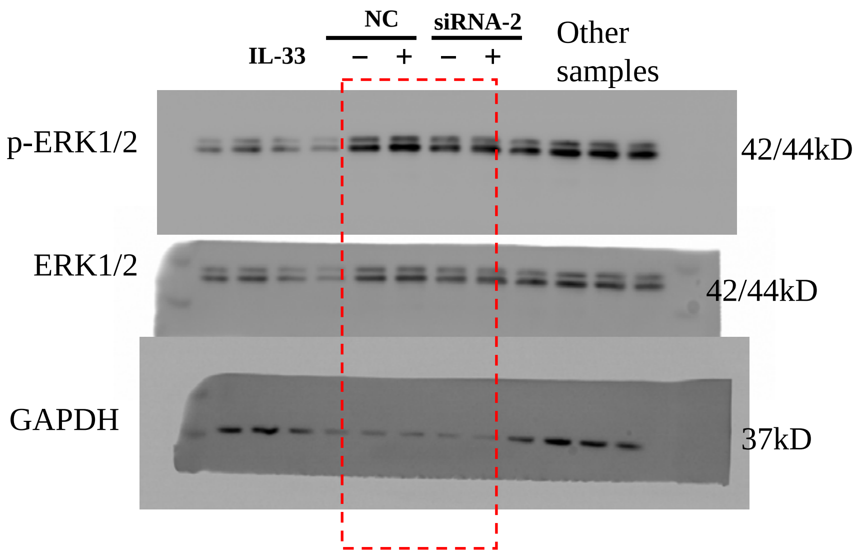

Full unedited gel for Figure 7K

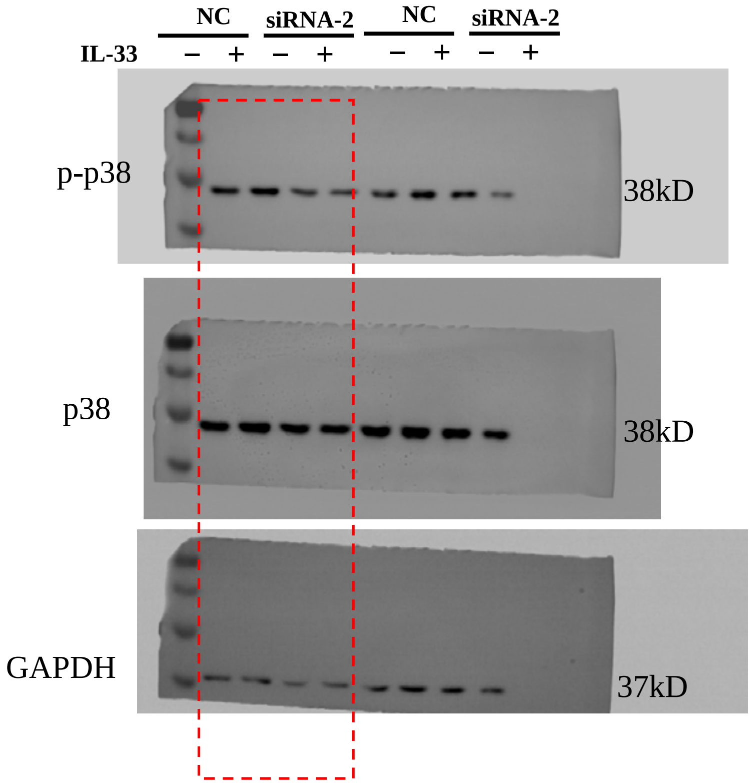

Full unedited gel for Figure 7L

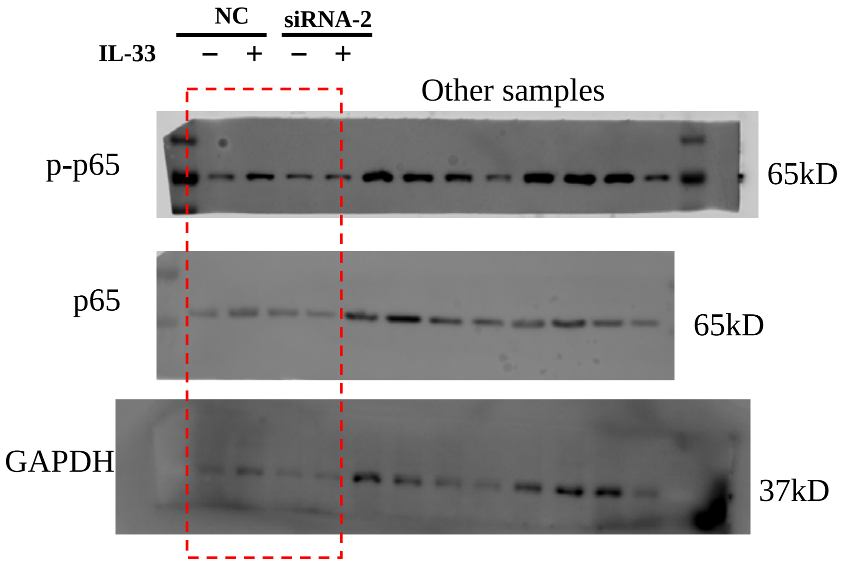

Full unedited gel for Figure 8C

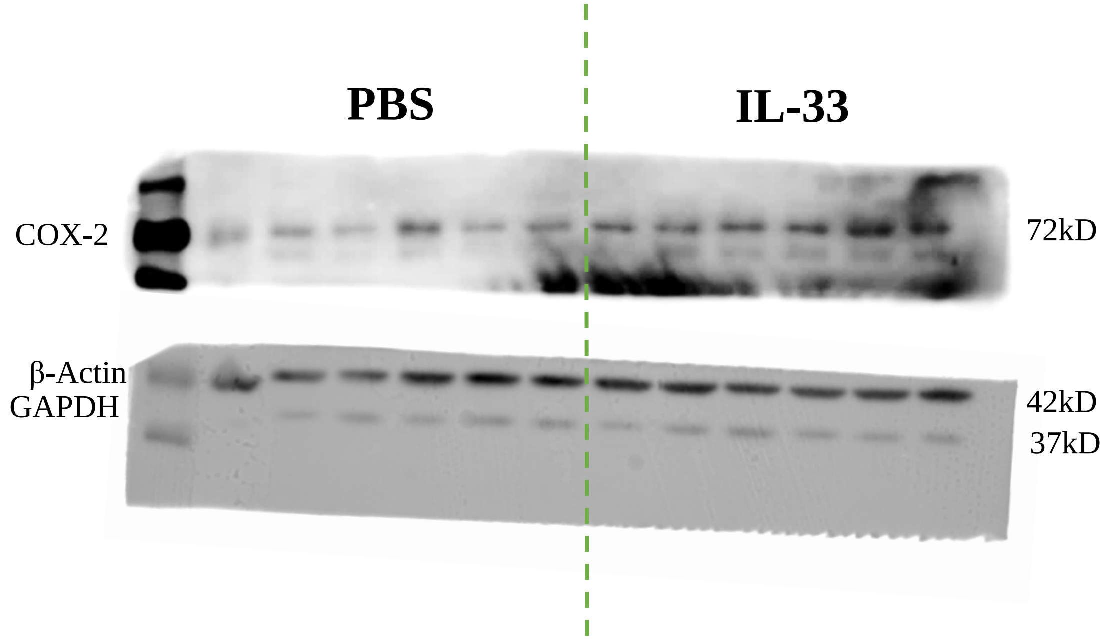

Full unedited gel for Supplementary Figure S1

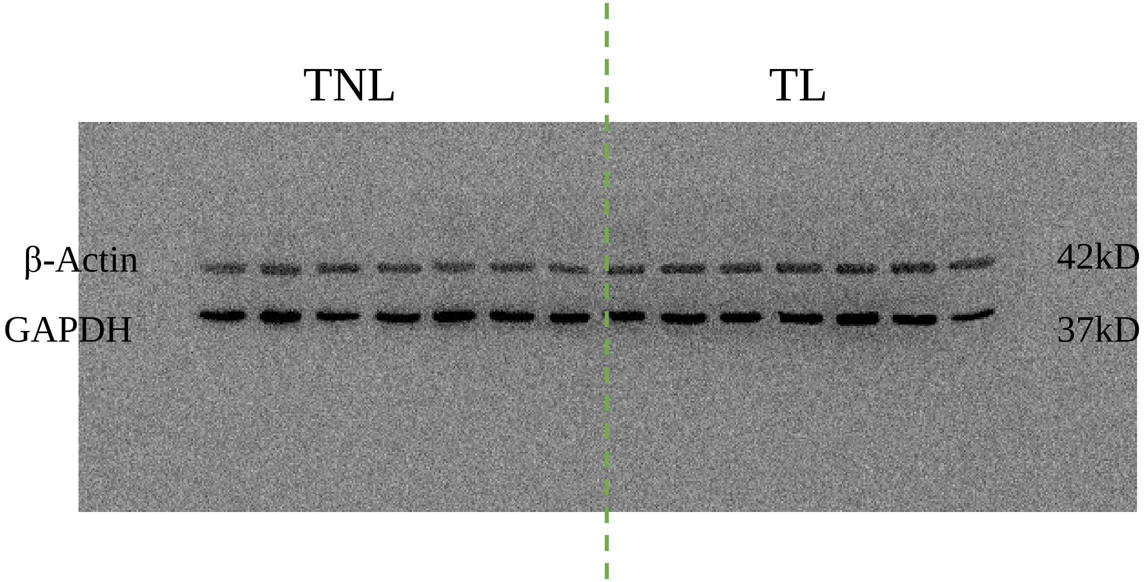

Full unedited gel for Supplementary Figure S2

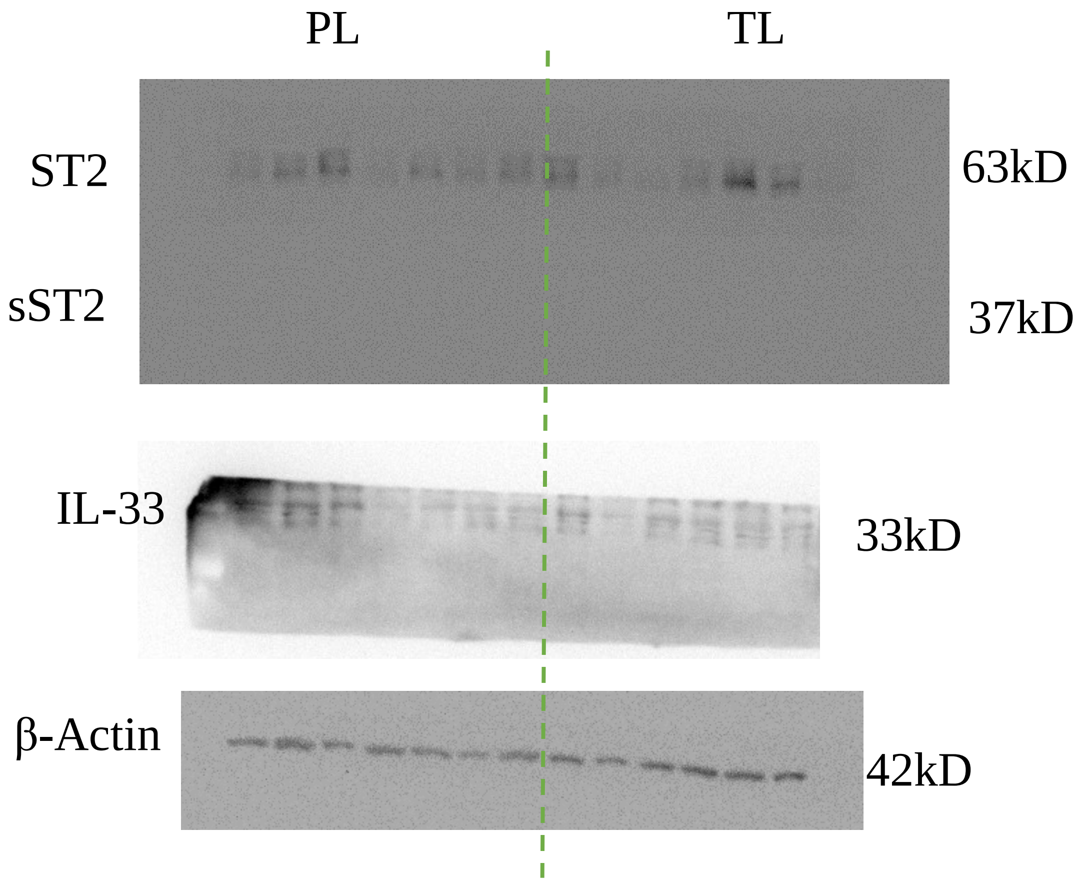

Supplement: Supplementary file 2 — Supplementary Material 2 [file 10020_2023_668_MOESM2_ESM.pdf]
